# Supplementary material for: Increasing uptake of FIT colorectal screening: protocol for the TEMPO randomised controlled trial testing a suggested deadline and a planning tool
Source: BMJ Open. 2023 May 18;13(5):e066136. doi: 10.1136/bmjopen-2022-066136 (PMC10201271; doi:10.1136/bmjopen-2022-066136)
Supplement: Supplementary data [file bmjopen-2022-066136supp001.pdf]

TIPS FOR USING YOUR KIT

Here are some tips that people find helpful to make the bowel screening kit even easier. Try drawing a line from any concern you have to a tip which might help you. You can draw as many or as few lines as you like. There are no right or wrong answers.

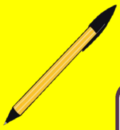

Concerns

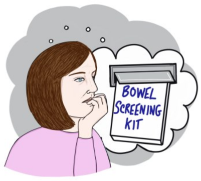

If I feel scared about bowel screening... ●

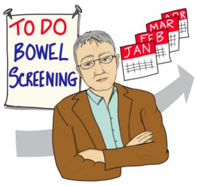

If I keep putting off using the kit... ●

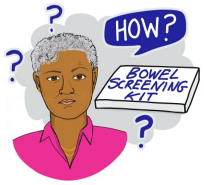

If I am not used to using a kit like this... ●

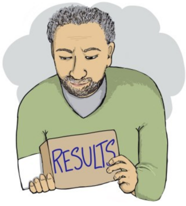

If I am worried what it might find... ●

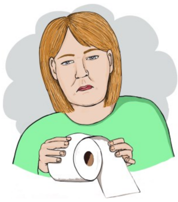

If I think using the kit is messy... ●

Do you have another concern? Please write it here: *If I…* ●

____

____

____

Tips

● … then I'll read the instructions.

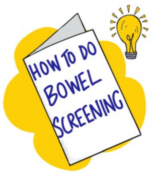

● … then I'll think that this kit could help save my life.

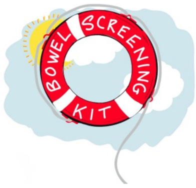

● … then I'll put the kit by the toilet to remind me.

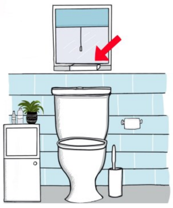

● … then I'll wash my hands after using the kit.

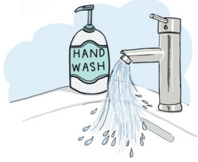

● ...then I'll tell myself that I'm responsible for my health.

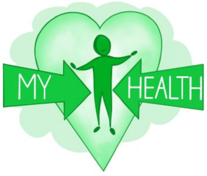

● Got a better tip? Please write it here: ...then *I'll…*

____

____

____
