# Supplementary material for: Increasing uptake of FIT colorectal screening: protocol for the TEMPO randomised controlled trial testing a suggested deadline and a planning tool
Source: BMJ Open. 2023 May 18;13(5):e066136. doi: 10.1136/bmjopen-2022-066136 (PMC10201271; doi:10.1136/bmjopen-2022-066136)
Supplement: Supplementary data [file bmjopen-2022-066136supp002.pdf]

Control group **i (no deadline, no planning sheet)**
FIT completers and FIT non-completers

Topic Guide

People's views on bowel screening

1. Experience of completing the FIT kit
   A few months ago you received a test kit like this in the post. Thinking back, can you tell me exactly what you did?
   - Did you have a clear plan about if/when/where/how you would do the test?
     - was it something you decided/did automatically/without thinking?
   - Was there anything that you found hard or difficult about doing the test?
     - *Prompt with barriers (forgetting, disgusting, embarrassing, worry, afraid of results, tempting fate, lack of time, unsure how to do)*
       - *Did you plan to overcome these?*

2. Acceptability of deadlines
   It has been suggested that giving people a suggested deadline for returning the bowel screening kit would be helpful. For example, people would be asked to return the kit within 1 week or 2 weeks or 4 weeks.
   - Do you think this would be acceptable? Would particular deadlines be more or less acceptable/effective? What would be your preference?
   - Would you like or dislike being a given a suggested deadline?
   - Do you think it is fair to peole to give a suggested deadline for returning the bowel screening kit?
   - Is it clear to you how being given a suggested deadline for returning the kit would be helpful in completing the kit?

3. **Acceptability of planning sheet**
   It has been suggested that asking people to link their concerns with tips for the bowel screening kit would be helpful. For example, people would be given a page with concerns and tips for using their bowel screening kit and asked to draw a line from a concern they have to a tip which might help them.
   - **Do you think this would be acceptable? Would particular concerns/ tips be more or less acceptable/effective? What would be your preference?**
   - **Would you like or dislike being being asked to link concerns and tips for your bowel screening kit?**
   - **Do you think it is fair to ask peole to link concerns and tips for their bowel screening kit?**
   - **Is it clear to you how being asked to link concerns and tips for your bowel screening kit would be helpful in completing the kit? Would you find it helpful, in what way?**

Robb KA, *et al. BMJ Open* 2023; 13:e066136. doi: 10.1136/bmjopen-2022-066136

Intervention groups **ii-iv (1-, 2-, 4-week deadline, no planning sheet)**
FIT completers and FIT non-completers

Topic Guide

People's views on bowel screening

1. Experience of completing the FIT kit

   A few months ago you received a test kit like this in the post. Thinking back, can you tell me exactly what you did?

   - Did you have a clear plan about if/when/where/how you would do the test?
     - was it something you decided/did automatically/without thinking?
   - Was there anything that you found hard or difficult about doing the test?
     - *Prompt with barriers (forgetting, disgusting, embarrassing, worry, afraid of results, tempting fate, lack of time, unsure how to do)*
       - *Did you plan to overcome these?*

2. Acceptability of deadlines

   Did you notice you were asked to return the kit by a certain time (1week/2 weeks/4 weeks) ?

   - Did you think this was acceptable? Would particular deadlines be more or less acceptable/effective? What would be your preference?
   - Did you like or dislike being a given a suggested deadline?
   - Do you think it is fair to peole to give a suggested deadline for returning the bowel screening kit?
   - Is it clear to you how being given a suggested deadline for returning the kit would be helpful in completing the kit? Did you find it helpful, in what way?

3. **Acceptability of planning sheet**

   It has been suggested that asking people to link their concerns with tips for the bowel screening kit would be helpful. For example, people would be given a page with concerns and tips for using their bowel screening kit and asked to draw a line from a concern they have to a tip which might help them.

   - **Do you think this would be acceptable? Would particular concerns/ tips be more or less acceptable/effective? What would be your preference?**
   - **Would you like or dislike being being asked to link concerns and tips for your bowel screening kit?**
   - **Do you think it is fair to ask peole to link concerns and tips for their bowel screening kit?**
   - **Is it clear to you how being asked to link concerns and tips for your bowel screening kit would be helpful in completing the kit? Would you find it helpful, in what way?**

Robb KA, *et al. BMJ Open* 2023; 13:e066136. doi: 10.1136/bmjopen-2022-066136

Intervention group v (no deadline, with a planning sheet)
FIT completers and FIT non-completers

Topic Guide

People's views on bowel screening

1. **Experience of completing the FIT kit**
   A few months ago you received a test kit like this in the post. Thinking back, can you tell me exactly what you did?
   - **Did you have a clear plan about if/when/where/how you would do the test?**
     - **was it something you decided/did automatically/without thinking?**
   - **Was there anything that you found hard or difficult about doing the test?**
     - ***Prompt with barriers (forgetting, disgusting, embarrassing, worry, afraid of results, tempting fate, lack of time, unsure how to do)***
       - ***Did you plan to overcome these?***

2. **Acceptability of deadlines**
   It has been suggested that giving people a suggested deadline for returning the bowel screening kit would be helpful. For example, people would be asked to return the kit within 1 week or 2 weeks or 4 weeks.
   - **Do you think this would be acceptable? Would particular deadlines be more or less acceptable/effective? What would be your preference?**
   - **Would you like or dislike being a given a suggested deadline?**
   - **Do you think it is fair to peole to give a suggested deadline for returning the bowel screening kit?**
   - **Is it clear to you how being given a suggested deadline for returning the kit would be helpful in completing the kit?**

3. **Acceptability of planning sheet**
   Did you notice you were given a page with tips for using your bowel screening kit?
   - **Did you think this was acceptable? Would particular concerns/ tips be more or less acceptable/effective? What would be your preference?**
   - **Did you like or dislike being asked to link concerns and tips for your bowel screening kit?**
   - **Do you think it is fair to ask peole to link concerns and tips for their bowel screening kit?**
   - **Is it clear to you how being asked to link concerns and tips for your bowel screening kit would be helpful in completing the kit? Did you find it helpful, in what way?**

Intervention groups vi-viii (1-, 2-, 4-week deadline, with a planning sheet)
FIT completers and FIT non-completers

Topic Guide

People's views on bowel screening

1. **Experience of completing the FIT kit**
   A few months ago you received a test kit like this in the post. Thinking back, can you tell me exactly what you did?
   - **Did you have a clear plan about if/when/where/how you would do the test?**
     - **was it something you decided/did automatically/without thinking?**
   - **Was there anything that you found hard or difficult about doing the test?**
     - ***Prompt with barriers (forgetting, disgusting, embarrassing, worry, afraid of results, tempting fate, lack of time, unsure how to do)***
       - ***Did you plan to overcome these?***

2. **Acceptability of deadlines**
   Did you notice you were asked to return the kit by a certain time (1week/2 weeks/4 weeks) ?
   - **Did you think this was acceptable? Would particular deadlines be more or less acceptable/effective? What would be your preference?**
   - **Did you like or dislike being a given a suggested deadline?**
   - **Do you think it is fair to peole to give a suggested deadline for returning the bowel screening kit?**
   - **Is it clear to you how being given a suggested deadline for returning the kit would be helpful in completing the kit? Did you find it helpful, in what way?**

3. **Acceptability of planning sheet**
   Did you notice you were given a page with tips for using your bowel screening kit?
   - **Did you think this was acceptable? Would particular concerns/ tips be more or less acceptable/effective? What would be your preference?**
   - **Did you like or dislike being asked to link concerns and tips for your bowel screening kit?**
   - **Do you think it is fair to ask peole to link concerns and tips for their bowel screening kit?**
   - **Is it clear to you how being asked to link concerns and tips for your bowel screening kit would be helpful in completing the kit? Did you find it helpful, in what way?**

Robb KA, *et al. BMJ Open* 2023; 13:e066136. doi: 10.1136/bmjopen-2022-066136
