# Supplementary material for: Increasing uptake of FIT colorectal screening: protocol for the TEMPO randomised controlled trial testing a suggested deadline and a planning tool
Source: BMJ Open. 2023 May 18;13(5):e066136. doi: 10.1136/bmjopen-2022-066136 (PMC10201271; doi:10.1136/bmjopen-2022-066136)
Supplement: Supplementary data [file bmjopen-2022-066136supp003.pdf]

University of Glasgow
School of Health & Wellbeing

NHS SCOTLAND

Mental Health and Wellbeing, Gartnavel Royal Hospital, Administration Building, 1st Floor,
1055 Great Western Road, Glasgow, G12 0XH

CONSENT FORM

Title of project: **People's views on bowel screening – interview study**

Name of Researchers: Dr Ben Young and Prof Katie Robb

Please initial box

1. I confirm that I have read and understand the information sheet Version 1.3 dated 5.8.2022 for the above study. ☐
2. I have had the opportunity to consider the information, ask questions and have had these answered satisfactorily ☐
3. I agree to the interview being audio-recorded and to its contents being used for research purposes. ☐
4. I understand that my participation is voluntary and that I am free to withdraw at any time, without giving any reason, without my medical care or legal rights being affected. ☐
5. I understand that my medical notes and data collected from the study may be looked at by regulatory authorities or by persons from NHS Scotland where it is relevant to my taking part in this study. I agree to these persons having access to this information. ☐
6. I agree to take part in the above study. ☐

______________________	______________________	______________________
Name of participant	Date	Signature
______________________	______________________	______________________
Researcher	Date	Signature

1 original for participant, 1 original for researcher

Robb KA, *et al. BMJ Open* 2023; 13:e066136. doi: 10.1136/bmjopen-2022-066136
