# Supplementary material for: Increasing uptake of FIT colorectal screening: protocol for the TEMPO randomised controlled trial testing a suggested deadline and a planning tool
Source: BMJ Open. 2023 May 18;13(5):e066136. doi: 10.1136/bmjopen-2022-066136 (PMC10201271; doi:10.1136/bmjopen-2022-066136)
Supplement: Author's manuscript [file bmjopen-2022-066136.draft_revisions.pdf]

BMJ Open is committed to open peer review. As part of this commitment we make the peer review history of every article we publish publicly available.

When an article is published we post the peer reviewers' comments and the authors' responses online. We also post the versions of the paper that were used during peer review. These are the versions that the peer review comments apply to.

The versions of the paper that follow are the versions that were submitted during the peer review process. They are not the versions of record or the final published versions. They should not be cited or distributed as the published version of this manuscript.

BMJ Open is an open access journal and the full, final, typeset and author-corrected version of record of the manuscript is available on our site with no access controls, subscription charges or pay-per-view fees (http://bmjopen.bmj.com).

If you have any questions on BMJ Open's open peer review process please email info.bmjopen@bmj.com

BMJ Open

Increasing uptake of FIT colorectal screening: protocol for the TEMPO randomised controlled trial testing a suggested deadline and a planning tool

Journal:	*BMJ Open*
Manuscript ID	bmjopen-2022-066136
Article Type:	Protocol
Date Submitted by the Author:	28-Jun-2022

Complete List of Authors:	Robb, Katie; University of Glasgow<br>Kotzur, Marie; University of Glasgow, Institute of Health & Wellbeing<br>Young, Ben; University of Glasgow<br>McCowan, Colin ; University of St. Andrews<br>Hollands, Gareth; University College London<br>Irvine, Audrey; NHS Tayside<br>MacDonald, Sara; University of Glasgow<br>McConnachie, Alex; University of Glasgow<br>O'carroll, Ronan; University of Stirling<br>O'Connor, Rory; University of Glasgow, Institute of Health and Wellbeing<br>Steele, Robert; University of Dundee, Surgery and Molecular Oncology

Keywords:	Gastrointestinal tumours < ONCOLOGY, PUBLIC HEALTH, PREVENTIVE MEDICINE

SCHOLARONE™
Manuscripts

Increasing uptake of FIT colorectal screening: protocol for the TEMPO randomised controlled trial testing a suggested deadline and a planning tool

Kathryn A. Robb*—Institute of Health and Wellbeing, University Of Glasgow, Glasgow, UK

Marie Kotzur—Institute of Health & Wellbeing, University Of Glasgow, Glasgow, UK

Ben Young—Institute of Health & Wellbeing, University Of Glasgow, Glasgow, UK

Colin McCowan—School of Medicine, University Of St. Andrews, St. Andrews, UK

Gareth J. Hollands—EPPI-Centre, UCL Social Research Institute, University College London, London, UK

Audrey Irvine — Scottish Bowel Screening Centre, NHS Tayside, Dundee, UK

Sara Macdonald—Institute of Health & Wellbeing, University Of Glasgow, Glasgow, UK

Alex McConnachie— Institute of Health & Wellbeing, University Of Glasgow, Glasgow, UK

Ronan E. O'Carroll—Psychology, University of Stirling, Stirling, UK

Rory C. O'Connor— Institute of Health & Wellbeing, University Of Glasgow, Glasgow, UK

Robert J.C. Steele—School of Medicine, University of Dundee, Dundee, UK

* corresponding author: Prof Kathryn A. Robb| Institute of Health and Wellbeing | Admin Building | Gartnavel Royal Hospital | 1055 Great Western Road | Glasgow G12 0XH; Katie.robb@glasgow.ac.uk; +44 (0)141 211 0685.

Word count: 3819

For peer review only

Abstract

Introduction: Screening can reduce deaths from colorectal cancer (CRC). Despite high levels of public enthusiasm, participation rates in population CRC screening programmes internationally remain persistently below target levels. Simple behavioural interventions such as completion goals and planning tools may support participation among those inclined to be screened but who fail to act on their intentions. This study aims to evaluate the impact of: a) a suggested deadline for return of the test and; b) a planning tool on return of faecal immunochemical tests (FITs) for CRC screening.

Methods and analysis: A randomised controlled trial of 40,000 adults invited to participate in the Scottish Bowel Screening Programme will assess the individual and combined impact of the interventions. Trial delivery will be integrated into the existing CRC screening process. The Scottish Bowel Screening Programme mails FITs to people aged 50-74 with brief instructions for completion and return. Participants will be randomised to one of eight groups: 1) no intervention; 2) suggested deadline (1-week); 3) suggested deadline (2-weeks); 4) suggested deadline (4-weeks); 5) planning tool; 6) planning tool plus suggested deadline (1-week); 7) planning tool plus suggested deadline (2-weeks); 8) planning tool plus suggested deadline (4-weeks). The primary outcome is return of the correctly completed FIT. To understand the cognitive and behavioural mechanisms and to explore the acceptability of both interventions, we will survey (n=2000) and interview (n=40) a subgroup of trial participants.

Ethics and dissemination: The study has been approved by the National Health Service South Central—Hampshire B Research Ethics Committee (ref. 19/SC/0369). The findings will be disseminated through conference presentations and publication in peer-reviewed journals. Participants can request a summary of the results.

Trial registration The trial is registered prospectively at clinicaltrials.gov (Identifier: NCT05408169, registration date: 07.06.2022).

Article summary

Strengths and limitations of this study

1. A 2x4 factorial RCT design will allow us to test the individual and combined impact of two behavioural interventions on colorectal screening uptake.
2. Integrating the RCT delivery into the existing colorectal screening process will provide evidence of the feasibility of providing either or both interventions at scale.
3. Cross-sectional and qualitative evaluation research will provide further insight into the psychological mechanisms that underlie any intervention effects.

Keywords (3-10) colorectal cancer; screening; faecal immunochemical test; deadline; planning; randomised controlled trial; qualitative interview

For peer review only - http://bmjopen.bmj.com/site/about/guidelines.xhtml

BACKGROUND

Colorectal cancer (CRC) is the second leading cancer killer in Scotland and worldwide.[1,2] Screening has been shown to reduce deaths from CRC if enough people invited participate.[3,4] The challenge is that high uptake is hard to achieve, and remains persistently below the target levels internationally. For example, screening rates are 39% in Australia and 62% in the USA.[5,6] Following the introduction of the single-sample faecal immunochemical test (FIT) in 2017, screening uptake in Scotland has risen to 65%, thus exceeding the Scottish target rate of 60%,[7] but remains below the internationally accepted European Union target uptake rate of at least 66%.[8] Although this is higher than the European population-based CRC screening programmes average of 25%,[8] large numbers of the population still do not screen and uptake is unevenly distributed across socioeconomic factors leading to health inequalities.[7]

Theoretical rationale

CRC screening uptake remains sub-optimal, despite high levels of public enthusiasm. In a Scottish survey, 85% of people reported intending to complete the FIT,[9] yet participation is 65%.[7] This suggests that apparent enthusiasm for the new FIT is not being translated into uptake. A major reason for this disparity may be that people are 'not getting round to it',[10] reflecting the well-recognised intention-behaviour gap and consistent with the observation that much of our behaviour is determined by factors other than conscious, deliberative control.[11-14] This disparity is consistent with previous work on 'inclined abstainers',[13] showing that a proportion of people are inclined to participate in screening but fail to do it. This explanation seems particularly likely for CRC screening which, unusually for a screening test, is self-completed at home. General open-ended intentions to act are often insufficient for action.[15] Our previous research has found that people often put off or forget to complete and return the FIT.[16] Additional support through prompting the setting of FIT completion goals and planning may better support participation among those inclined to complete the FIT but who might otherwise fail to act.

For peer review only

Goal setting

Goal setting, and particularly action-planning, can help initiate behaviour in those inclined to act. This is achieved by prompting a person to plan when, where and how to act, which lessens the decision-making burden when that situation is encountered.[17-19] For example, a suggested deadline by which a task should be completed can be an implicit prompt to undertaking action planning. Currently, CRC screening invitations in Scotland provide detailed information about 'where' and 'how to act' but 'when to act' is left open, potentially reducing the likelihood that recipients will plan and act accordingly. In breast and cervical screening, providing a fixed appointment time increased uptake compared to an open invitation.[20] Similarly, in our recent qualitative study, women who attended breast and cervical screening but had not completed CRC screening described how the lack of an appointment facilitated the delay and forgetting of CRC screening: "If it's a bowel screening one, yep, put it somewhere and think 'Yes, I'll do that' and then forget about it because it doesn't have an appointment date. I think if something has an appointment date, you're forced to act." (54

years, not screened for CRC).[16(p.6)] Recent evidence suggests that both short (1-week) and long (3-week) deadlines achieve small but significant increases in FIT return.[21] Optimal deadline length, however, has yet to be determined. It is important to establish if a suggested deadline for FIT kit return can support CRC screening participation compared to an open invitation in a population-based screening programme.

Planning support

Systematic reviews clearly demonstrate that interventions supporting people to plan how to enact a behaviour (including action and coping planning) are particularly suited to changing the behaviour of inclined abstainers.[12] Indeed, planning support interventions have been shown to be more effective than other approaches, such as self-monitoring or providing instructions,[22] and have high adherence rates.[23] Planning interventions are often based on implementation intentions which promote behaviour change using 'if-then' plans and have successfully reduced alcohol consumption and smoking, and increased cancer screening attendance and physical activity.[11,12,24,25] Previous research on implementation intention interventions for CRC screening has had limited success when it has relied on prepared statements of barriers ('if') and possible solutions ('then').[26] Encouraging people to form their own implementation intentions for CRC screening appears to produce clinically significant increases in uptake.[24] Planning tools (also called volitional help sheets [VHS]) emphasise this by requiring users to physically draw a line to link a barrier e.g. "If I am tempted to eat when I am anxious" with a solution e.g. "then I will reward myself when I do not overeat." to ensure user-engagement.[27(p.706)] The rationale is that the 'if' barriers or critical situations are made salient and, as a result, the 'then' response or solution comes automatically to mind.

The Medical Research Council's recently updated guidance on complex intervention development and evaluation recommends addressing questions beyond intervention effectiveness, such as "whether and how the intervention will be acceptable, implementable, cost effective, scalable, and transferable across contexts.".[28(p.2)] With a view to implementing a suggested deadline or planning tool at national scale, if effective, the acceptability of both interventions to CRC screening service users must be investigated.

Objectives

The overall aim of the TimE fraMe and Planning tOol (TEMPO) study is to evaluate the impact of a suggested deadline and planning tool on FIT return in the Scottish Bowel Screening Programme.

The study addresses six research questions:

1. Does providing a suggested deadline increase uptake of CRC screening?
2. Does providing a FIT planning tool increase uptake of CRC screening?
3. Does providing both a suggested deadline and a FIT planning tool increase uptake of CRC screening?
4. Does the length of the suggested deadline (one, two or four weeks) impact upon uptake of CRC screening?

For peer review only - http://bmjopen.bmj.com/site/about/guidelines.xhtml

5. What are the cognitive and behavioural mechanisms underlying any observed effect of providing a suggested deadline, a planning tool, or a combination of both?
6. How acceptable do participants find receiving a suggested deadline, a planning tool, or a combination of both?

METHODS AND ANALYSIS

The study employs a 2x4 factorial randomised controlled trial (RCT) design combined with cross-sectional and qualitative evaluation research with a sub-sample of trial participants. Figure 1 shows the CONSORT flow chart.

Study setting

This is a single-centre trial to be conducted in collaboration with the Scottish Bowel Screening Centre based in Dundee, Scotland, and integrated into the national CRC screening programme.

Eligibility criteria

All people eligible for CRC screening in Scotland who have a Community Health Index Number can be included in this study.

Recruitment and allocation

Following established recruitment procedures,[29,30] working in partnership with the Scottish Bowel Screening Centre staff, 40,000 consecutive adults due to be sent a FIT will be randomised to one of eight groups (Figure 1) from 20.06.2022 over approximately 10 days.

The randomisation schedule will be computer-generated at the Robertson Centre for Biostatistics, University of Glasgow. The schedule will be stored in a secure area of the Robertson Centre for Biostatistics network, with no access for those developing the statistical analysis programs and transferred via a secure web portal to the Scottish Bowel Screening Centre team, who identify when people are to be invited for CRC screening. The IT system electronically generates the appropriate mailing packages to be sent out to each person. For this study, randomisation will be performed by the method of randomised permuted blocks of length 8, so that in every 8 packages sent, exactly one of each type will be included.

Blinding

The process of generating and mailing the routine screening invitation letters is fully automated and is conducted by a large mail-handling company. A data file containing contact details for the screening invitation letters is sent to the mail-handling company daily. The company will print screening invitation letters with suggested deadlines and add the planning tool in accordance with the participants' allocation within this study. The researchers, including those performing the analysis, will therefore be blind to the allocation of participants. The participants will not be informed of the experiment.

Interventions

For peer review only - http://bmjopen.bmj.com/site/about/guidelines.xhtml

There will be eight allocation groups (Figure 1): Group 1 – standard invitation (n = 5,000); Group 2 – 1-week deadline (n=5,000); Group 3 – 2-week deadline (n=5,000); Group 4 – 4-week deadline (n=5,000); Group 5 – planning tool (n=5,000); Group 6 – planning tool plus 1-week deadline (n=5,000); Group 7 – planning tool plus 2-week deadline (n=5,000); Group 8 – planning tool plus 4-week deadline (n=5,000).

Deadline intervention

Those allocated to the deadline intervention (groups 2-4 and 6-8) will receive a screening invitation letter with one of three suggested deadlines added (Table 1): 1 week, 2 weeks and 4 weeks. This choice was based on feedback from the Cancer Research UK Early Diagnosis Advisory Group, public and patient involvement representatives, and observation of kit return patterns in the Scottish Bowel Screening Programme. The deadline will be displayed centrally on the standard CRC screening invitation letter as a bold, highlighted sentence (Table 1). The wording *"or as soon as possible"* is intended to make clear that the deadline constitutes a suggested return date only, to avoid discouraging participants who are unable to meet the deadline. The existing invitation letter already states "*post it as soon as possible*" and the suggested deadline builds on this by providing a more explicit goal. Furthermore, a standard reminder will be sent after 6 weeks without a deadline and so will provide a safety net for any potential participants who have missed the deadline in their first invitation letter.

Table 1 Deadline intervention arms

Intervention group	Suggested FIT return deadline	Deadline sentence
Groups 2 and 6	1-week deadline	Please return your kit within 1 week (by [insert date 10 days from posting date]) or as soon as possible.
Groups 3 and 7	2-week deadline	Please return your kit within 2 weeks (by [insert date 17 days from posting date]) or as soon as possible.
Groups 4 and 8	4-week deadline	Please return your kit within 4 weeks (by [insert date 31 days from posting date]) or as soon as possible.

Planning tool intervention

Those allocated to the planning tool intervention (groups 5-8) will receive a planning tool with their screening invitation. The planning tool will be presented on a separate single sheet of paper inserted into the CRC screening invitation envelope. It asks participants to identify "*concerns*" (i.e. barriers) they have with using the FIT from a list and to link them to a "*tip*" (i.e. solution) from an adjacent list to help them overcome this concern. The planning tool reads, *"Here are some tips that people find helpful to make the bowel screening kit even easier. Try drawing a line from any concern you have to a tip which might help you. You can draw as many or as few lines as you like. There are no right or wrong answers."* Concerns reflect critical situations in conventional planning tool designs and tips reflect solutions

For peer review only - http://bmjopen.bmj.com/site/about/guidelines.xhtml

(Armitage et al., 2014). Each concern and tip is accompanied by an image illustrating its meaning. Finally, participants will have the option to write their own concern or tip. While the planning tool instructions encourage people to use the planning tool, they do not suggest that participants must do so in order to take part in CRC screening. The specific listed concerns and tips are based on extensive development work involving consideration of existing theoretical and empirical literature, in-depth qualitative interviews, a survey of people who had been invited to complete a FIT, and co-design workshops to create a user-friendly planning tool with members of the public who have completed the FIT and others who have not completed the FIT.[31-33] The final planning tool was reviewed and approved by the Research Team, Patient and Public Involvement representatives, and a patient and public group of the Scottish Primary Care Network.

Table 2 displays the concerns and tips shown in the planning tool. The planning tool is presented in Supplementary file 1.

Table 2 Planning tool intervention—concerns and tips

Concerns	Tips
If I feel scared about bowel screening…	… then I'll read the instructions.
If I keep putting off using the kit…	… then I'll think this this kit could help save my life.
If I am not used to using a kit like this…	… then I'll put the kit by the toilet to remind me.
If I am worried what it might find…	… then I'll wash my hands after using the kit.
If I think using the kit is messy…	… then I'll tell myself that I'm responsible for my health.

Control group

Groups 1 and 5 together make up the control group for the assessment of suggested deadlines. Groups 1-4 make up the control group for assessment of the planning tool. Only participants in Group 1 will be sent a standard CRC screening invitation and FIT (without a suggested deadline or a planning tool) according to normal current practice.

It is also important to note that three groups will receive both a planning tool and a suggested deadline, which will allow assessments of the combined effects of a suggested deadline and the planning tool compared to receiving a deadline only, a planning tool only, or neither.

Outcomes

Our primary outcome is the proportion of FITs returned correctly completed to be tested by the CRC screening laboratory providing a positive or negative result, within three months of the FIT being sent to a person. The return and successful testing of any replacement FITs that were sent to people after the first FIT at study baseline will be treated as a FIT return. As secondary outcomes, we will evaluate cognitive and behavioural mechanisms and explore the acceptability of both interventions.

Sample size

This 2x4 factorial RCT involves the assessment of two interventions, so each will be assessed at a significance level of 2.5%, to preserve an overall Type I error rate of 5%.

The assessment of the effect of the planning tool will involve the comparison of two groups of 20,000 participants. At a significance level of 2.5%, this gives 90% power to detect an

For peer review only - http://bmjopen.bmj.com/site/about/guidelines.xhtml

increase in uptake rates from 65% to 6.68%. Alternatively, if the intervention increases uptake rates from 60% to 68%, the 97.5% confidence interval for the difference in uptake rates will have a width of ±1.06%.

The assessment of the effect of suggested deadlines for FIT kit return will involve four groups of 10,000 participants. Comparing each suggested deadline with the control group (no deadline) at a significance level of 0.83% (one third of 2.5%), there will be 90% power to detect increases in uptake rates from 65% to 67.63%. If an intervention increases uptake from 65% to 68%, the 99.17% confidence interval for the difference in uptake rates will have a width of ±1.76%.

Trial data collection and management

For the purposes of this study, two additional variables will be added to the Scottish Bowel Screening Programme invitation data file: i) a unique identifier for each participant; and ii) a variable coding for the allocated condition. The pseudonymised participant data including FIT kit return data will be transferred from the Scottish Bowel Screening Centre in Dundee to the Robertson Centre for Biostatistics Analytical Platform at the University of Glasgow using a secure file transfer protocol. The Robertson Centre for Biostatistics Analytical Platform provides a secure analytical platform with access only available to the approved research team who can analyse the data but cannot remove or copy the data. Results of analyses must go through a disclosure control process to prevent potentially identifiable information being released, in order to be extracted from the platform.

Data analysis plan

Primary outcome

The primary analysis will use a logistic regression model, including a binary variable for whether the planning tool was provided, and a four-level categorical variable denoting which deadline was suggested. Age, sex, and area deprivation (measured by the Scottish Index of Multiple Deprivation [SIMD][34]) will be included in the model, to improve model specification. The main effects of each intervention will be reported as an odds ratio, with a confidence interval (CI) and p-value. For the estimate of the effect of the planning tool, a 97.5% CI will be reported. For estimates of the effect of alternative suggested deadlines, relative to no deadline, 99.17% CIs will be used. Subsequent analyses will model the interaction between the two interventions, to assess whether the interventions have independent effects or are synergistic. We shall also model interactions between each intervention effect and age, sex, and deprivation, to assess whether the interventions have different effects for subgroups of the population. Assessing uptake by deprivation is important given the disparities in screening uptake by deprivation (53% most deprived versus 73% least deprived;[7]) which any intervention could reduce or exacerbate.[35]

Secondary outcomes

We will conduct two studies to evaluate secondary outcomes, answering research questions 5 and 6.

Study 2

For peer review only - http://bmjopen.bmj.com/site/about/guidelines.xhtml

We will conduct a questionnaire-based case-control study to assess the cognitive and behavioural mechanisms underlying any effect of providing a suggested deadline for FIT kit return and a planning tool.

Recruitment. At least three months after the screening invitation, a subsample of trial participants will be identified from all eight groups, including people who returned and did not return their FIT, and people living in 40% most and least deprived neighbourhoods (based on SIMD quintiles). The subsample will be sent a postal invitation to complete and return a questionnaire. The aim is to obtain responses from approximately 1,000 people who have returned the FIT and 1,000 who have not returned the FIT, across the eight groups. Based on previous work, we anticipate achieving a higher response rate from those returning the FIT and a lower response from those not returning the FIT, therefore approximately 11,872 questionnaires will be mailed, as shown in Figure 1.[32] A reminder questionnaire will be sent to those who have not returned the questionnaire after three weeks.

Power analysis. The study is a case-control study (1,000 cases, 1,000 controls). The study will have 90% power at a 5% significance level to detect an odds ratio for being a case of 1.146 per standard deviation increase in a continuous predictor variable; for a binary predictor with 50% prevalence, the study will have 90% power to detect an odds ratio for being a case of 1.345; for a binary predictor with a prevalence of 10%, the study will have 90% power to detect an odds ratio for being a case of 1.662.

Materials and procedure. A brief questionnaire has been designed to assess cognitive and behavioural mechanisms, e.g. action and coping planning,[17] with items used previously adapted for the CRC screening context and acceptability using the theory-informed Acceptability Questionnaire,[19,36] as well as demographic characteristics. Participants will be asked if they are willing to participate in future research to assist recruitment for Study 3.

Analysis. Descriptive statistics, Chi-square tests, ANOVA and logistic regression will be used to examine cognitive and behavioural mechanism differences between the intervention and control groups and their association with FIT kit return.

Study 3

To explore the acceptability of a suggested deadline, a planning tool, and a combination of both, we will conduct a qualitative interview study.

Recruitment. Interview participants will be sampled from Study 2 participants who have indicated willingness to participate in further research. We will recruit approximately 40 participants and will aim to achieve a balance of participants who have and have not returned the FIT, men and women, from across the CRC screening invitation age range (50-74 years), from both deprived and affluent neighbourhoods (based on SIMD), and from the eight trial allocation groups.

Procedure. Potential participants will be contacted to request participation in a semi-structured qualitative interview conducted face-to-face, by telephone or video-call. Interviews will last approximately 30-60 minutes, and topic guides will focus on participants'

nly

For peer review only - http://bmjopen.bmj.com/site/about/guidelines.xhtml

experiences of being invited for FIT with and without a suggested FIT return deadline and a planning tool. Participants will be offered £30 towards travel and expenses.

Analysis. The interviews will be audio-recorded, transcribed verbatim and analysed using the Framework Method.[37] Themes from the questionnaire data in Study 2 will be explored in depth e.g. action and coping planning,[17] as well as constructs within the theoretical framework of acceptability.[38] Calls to the Scottish Bowel Screening Programme helpline relating to the suggested deadline for FIT kit return and planning tool will also be monitored as an additional assessment of acceptability.

Participant timeline

This protocol describes a three-month project as shown in Figure 2.

Study management and monitoring

This trial combines two individually funded studies with the same Principal Investigator. The trial will be managed by the co-investigator teams of both studies including the Principal Investigator, academic co-investigators, key researchers, and public and patient involvement representatives who will oversee trial conduct and progress together. Data management, participant confidentiality and the conduct of all trial staff will adhere to the protocol (Version 1.4, 06.09.2021 or subsequent approved versions), Good Clinical Practice guidelines, and the Data Protection Act 2018. An audit trail of all documentation and data collection will be kept in an electronic study site file to facilitate monitoring by the research team and the sponsor. This trial is sponsored by the NHS Greater Glasgow and Clyde. Amendments to the protocol will be notified to the sponsor and the responsible Research Ethics Committee for their approval. The sponsor may audit the trial at any time and without notice.

Stopping criteria

The CRC screening invitations and kits will be posted as one batch over approximately 10 days. There are no stopping criteria for recruitment.

Harms

This is a low-risk trial providing people eligible for the Scottish Bowel Screening Programme with additional written information. While we recognise that providing a suggested deadline or a planning tool may increase the burden of completing the FIT, we will ensure that participants: a) are not disadvantaged if they cannot meet the suggested deadline for returning the FIT; and b) are under no obligation to use the planning tool in order to complete CRC screening. We will be able to evaluate the additional burden of each intervention in evaluation studies 2 and 3.

Public and patient involvement

The study is supported by two patient and public representatives (PPRs) who have provided input throughout the programme of research. The PPRs were involved in the design of the study, the creation of materials to support the recruitment and join the co-investigator meetings to discuss study progress. At the end of the study, the PPRs will review a plain language summary of the study findings which we will make available to the interview participants.

For peer review only

Intervention materials for this study, i.e. the planning tool and the screening invitation letters including the three suggested deadlines, have been developed in workshops and focus groups and have received feedback from the PPRs, the Cancer Research UK Early Diagnosis Advisory Group, and the PPRs of the Scottish Primary Care Research network.

ETHICS AND DISSEMINATION

Ethical approval has been granted by the UK National Health Service South Central – Hampshire B Research Ethics Committee (REC ref. no. 19/SC/0369). The research will be carried out in accordance with the World Medical Association Declaration of Helsinki (1964) and its revisions (Tokyo [1975], Venice [1983], Hong Kong [1989], South Africa [1996] and Edinburgh[2000]).

The results from this study will be presented at national and international conferences and written up for publication in peer reviewed journals. The study is expected to produce two manuscripts: i) primary findings of the RCT and ii) the findings of the secondary evaluation. The co-authors have a track record of public and media engagement in research. They will work closely with the University Of Glasgow Press Office to disseminate the findings to the public. Study participants will receive a summary of the findings if they request this.

This trial will investigate the individual and combined impact of two innovative CRC screening interventions. Providing a suggested deadline and providing a FIT planning tool are low-cost interventions that would be easily implemented in routine practice. In addition, this study will provide evidence on the most effective timeframe for a suggested deadline as well as the cognitive and behavioural mechanisms that underlie any intervention effects. Finally, the study will investigate the acceptability of providing either intervention to those invited to participate in CRC screening.

DECLARATIONS

Acknowledgements
We thank the PPRs, Mary Cameron and Lucy J. Robertson, for their continued support of this research, the patient and public involvement group of the Scottish Primary Care Research Network for their feedback on the intervention materials, and Catherin McKay of ListenThinkDraw who provided illustrative services during the development of the planning tool and images for the final version.

Author contributions
KAR conceived both the planning tool and deadline interventions. KAR and MK designed the intervention procedures, study materials and evaluation with input from BY, SM, REOC, RCOC, AI, CMC, AM, GH and RJCS. AM conducted the sample size calculations and designed the statistical analyses. KAR submitted the original application for ethical approval and MK and BY drafted and managed approval amendments. KAR, BY and MK drafted the protocol manuscript. All co-authors read and approved the final manuscript.

For peer review only

Funding
This study is supported by an Early Diagnosis Advisory Group (EDAG) Project Grant from Cancer Research UK (C9227/A27877 - Increasing uptake of faecal immunochemical test (FIT) bowel screening: trial of providing a suggested deadline for FIT kit return, PI KAR, Co-investigators: CMC, AM, GJH, RJCS) and by a Response Mode Grant from the Scottish Chief Scientist Office (HIPS/17/23 - Increasing uptake of bowel cancer screening: development of a FIT planning support tool, PI: KAR, Co-investigators: SM, REOC, RCOC, AI, RJCS, MK). The funders have no involvement in the study design, data collection or analysis, or the writing and publication of reports of the study. All researchers involved in this study were independent of the funder.

Competing interests
The authors declare that they have no competing interests.

Consent for publication
Not applicable

Availability of data and materials
Not applicable

For peer review only

REFERENCES

1. Ferlay J, Ervik M, Lam F, et al. Cancer today (powered by GLOBOCAN 2018). IARC CancerBase. number: 15. [Internet]. n.d. [cited 2019 Dec 11]. Available from http://publications.iarc.fr/577.
2. Information Services Division. Cancer Mortality in Scotland: Annual update to 2018. [Internet]. 2019. [cited 2019 Dec 11] Available from https://www.isdscotland.org/Health-Topics/Cancer/Publications/2019-10-29/2019-10-29-Cancer-Mortality-Report.pdf?
3. Libby G, Brewster DH, McClements PL, et al. The impact of population-based faecal occult blood test screening on colorectal cancer mortality: a matched cohort study. *Br J Cancer*. 2012;107(2):255-9.
4. Steele RJ, McDonald PJ, Digby J, et al. Clinical outcomes using a faecal immunochemical test for haemoglobin as a first-line test in a national programme constrained by colonoscopy capacity. *United European Gastroentero J* 2013;1(3):198-205.
5. Kuipers EJ, Lansdorp-Vogelaar I. Colorectal cancer screening in Australia. *Lancet Public Health* 2017;2(7):e304-e5.
6. White A, Thompson TD, White MC, et al. Cancer screening test use—United States, 2015. *MMWR Morb Mortal Wkly Rep* 2017;66(8):201.
7. Public Health Scotland. Scottish Bowel Screening Programme Statistics: For the period of invitations between May 2019 and April 2021. [Internet]. 2022. [cited 2022 June 23] Available at https://publichealthscotland.scot/media/11605/2022-02-08-bowel-screening-publication-report.pdf.
8. Senore C, Basu P, Anttila A, et al. Performance of colorectal cancer screening in the European Union Member States: data from the second European screening report. *Gut* 2019;68(7):1232-44.
9. Chambers JA, Callander AS, Grangeret R, et al. Attitudes towards the Faecal Occult Blood Test (FOBT) versus the Faecal Immunochemical Test (FIT) for colorectal cancer screening: Perceived ease of completion and disgust. *BMC Cancer* 2016;16(1):96-102.
10. Lo SH, Waller J, Wardle J, et al. Comparing barriers to colorectal cancer screening with barriers to breast and cervical screening: A population–based survey of screening-age women in Great Britain. *J Med Screen* 2013;20(2):73-79.
11. Gollwitzer PM, Sheeran P. Implementation intentions and goal achievement: A meta-analysis of effects and processes. In: Advances in experimental social psychology, Vol. 38. San Diego: Elsevier Academic Press Inc. 2006:69-119.
12. Kwasnicka D, Presseau J, White M, et al. Does planning how to cope with anticipated barriers facilitate health-related behaviour change? A systematic review. *Health Psychol Rev* 2013;7(2):129-145.
13. Orbell S, Sheeran P. 'Inclined abstainers': A problem for predicting health-related behaviour. Br J Soc Psychol 1998;37(2):151-165.
14. Hollands GJ, Marteau TM, Fletcher PC. Non-conscious processes in changing health-related behaviour: A conceptual analysis and framework. *Health Psychol Rev* 2016;10(4):381–394.
15. Webb T, Sheeran P. Does changing behavioural intentions engender behaviour change: A meta-analysis of the experimental evidence. *Psychol Bull 2006;132:*249-268.

For peer review only - http://bmjopen.bmj.com/site/about/guidelines.xhtml

16. Kotzur M, McCowan C, Macdonald S, et al. Why colorectal screening fails to achieve the uptake rates of breast and cervical cancer screening: a comparative qualitative study. *BMJ Qua Saf* 2020;29(6):482-90.
17. Gollwitzer PM. Implementation intentions: Strong effects of simple plans. *Am Psychol* 1999;54(7): 493.
18. Michie S, Dormandy E, Marteau TM. Increasing screening uptake amongst those intending to be screened: the use of action plans. Patient Educ Couns 2004;55(2):218-22.
19. Presseau J, Johnston M, Heponiemi T, et al. Reflective and automatic processes in health care professional behaviour: a dual process model tested across multiple behaviours. *Ann Behav Med* 2014;48(3), 347-358.
20. Duffy SW, Myles JP, Maroni R, et al. Rapid review of evaluation of interventions to improve participation in cancer screening services. *J Med Screen* 2017;24(3): 127-145.
21. Lieberman, A., Gneezy A, Berry E. The effect of deadlines on cancer screening completion: a randomized controlled trial. *Sci Rep* 2021;11(1):13876.
22. Dombrowski SU, Sniehotta FF, Avenell A, et al. Identifying active ingredients in complex behavioural interventions for obese adults with obesity-related co-morbidities or additional risk factors for co-morbidities: A systematic review. *Health Psychol Rev* 2012;6(1):7-32.
23. O'Connor RC, Ferguson E, Scott F, et al. A randomised controlled trial of a brief psychological intervention to reduce repetition of self-harm in patients admitted to hospital following a suicide attempt. *Lancet Psychiatry* 2017;4(6):451-60.
24. Neter E, Stein N, Barnett-Griness O, et al. From the Bench to Public Health: Population-level Implementation Intentions in Colorectal Cancer Screening. *Am J Prev Med* 2014;46(3): 273-280.
25. Rutter DR, Steadman L, Quine L. An implementation intentions intervention to increase uptake of mammography. *Ann Behav Med* 2006;32(2):127-34.
26. Lo SH, Good A, Sheeran P, et al. Preformulated implementation intentions to promote colorectal cancer screening: A cluster-randomized trial. *Health Psychol* 2014;33(9):998-1002.
27. Armitage CJ, Norman P, Noor M, et al. Evidence that a very brief psychological intervention boosts weight loss in a weight loss program. *Behav Ther* 2014;45(5):700–707.
28. Skivington K, Matthews L, Simpson SA, et al. A new framework for developing and evaluating complex interventions: Update of Medical Research Council guidance. *BMJ* 2021;374.
29. Libby, G, Bray, J, Champion, J, et al. Pre-notification increases uptake of colorectal cancer screening in all demographic groups: a randomized controlled trial. *J Med Screen* 2011;8,24-29.
30. O'Carroll RE, Chambers JA, Brownlee L, et al. Anticipated regret to increase uptake of colorectal cancer screening (ARTICS): A randomised controlled trial. *Soc Sci Med*. 2015;142:118-127.
31. Kotzur M, Macdonald S, O'Connor R, et al. Identifying barriers and solutions to self-completed FIT bowel screening to develop a volitional help-sheet. 33rd Annual

For peer review only

For peer review only - http://bmjopen.bmj.com/site/about/guidelines.xhtml

Conference of the European Health Psychology Society; 2019 September 03-07; Dubrovnik, Croatia.

32. Kotzur M, Macdonald S, O'Connor R, et al. Assessing barriers to self-completed colorectal cancer screening and potential solutions: A cross-sectional study. International Congress of Behavioral Medicine; 2021 June 11-17; virtual congress.
33. Robb KA. Achieving solutions to inequalities in cancer screening. Cancer Research UK/UK Society for Behavioural Medicine Cancer Special Interest Group Behavioural Science Webinar; 2022 June 07; virtual seminar.
34. Scottish Government. Scottish Index of Multiple Deprivation 2020. [Internet]. [cited 2022 May 25]. Available from https://www.gov.scot/collections/scottish-index-of-multiple-deprivation-2020/
35. Lorenc T, Petticrew M, Welch V, et al. What types of interventions generate inequalities? Evidence from systematic reviews. *J Epidemiol Community Health* 2013;67:190-193.
36. Sekhon M, Cartwright M, Francis JJ. Development of a theory-informed questionnaire to assess the acceptability of healthcare interventions: Application of pre-validation methods. UK Society of Behavioural Medicine Annual Scientific Meeting; 2018 December 12-13; Birmingham, UK.
37. Ritchie J, Spencer L. Qualitative data analysis for applied policy research. In Ritchie J, Spencer L, Bryman A et al. eds. Analysing Qualitative Data. London, UK: Routledge 1994:173-194.
38. Sekhon M, Cartwright M, Francis JJ. Acceptability of healthcare interventions: an overview of reviews and development of a theoretical framework. *BMC Health Serv Res* 2017;17(1):88.

For peer review only

For peer review only - http://bmjopen.bmj.com/site/about/guidelines.xhtml

FIGURE TITLES AND LEGENDS

Figure 1 CONSORT flow chart

Figure 2 Participant timeline
Note. * denotes data collection end points

For peer review only

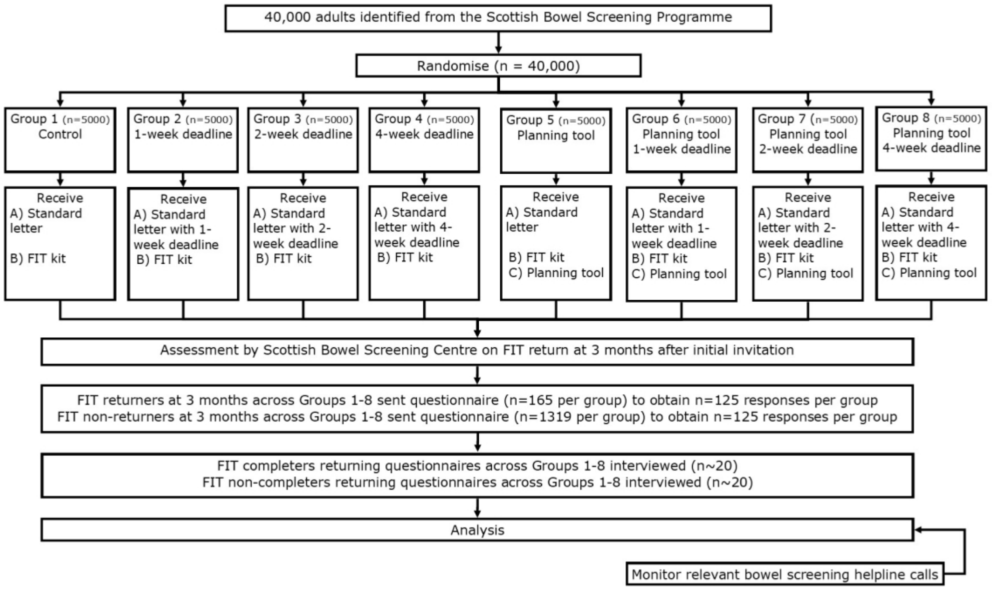

Figure 1 CONSORT flow chart

297x209mm (150 x 150 DPI)

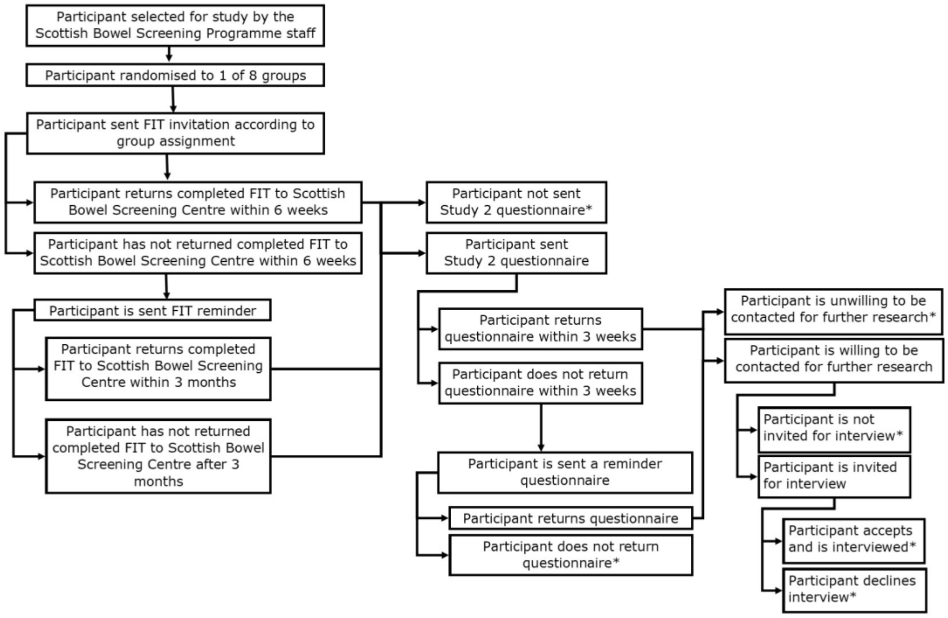

Figure 2 Participant timeline

297x209mm (150 x 150 DPI)

TIPS FOR USING YOUR KIT

Here are some tips that people find helpful to make the bowel screening kit even easier. Try drawing a line from any concern you have to a tip which might help you. You can draw as many or as few lines as you like. There are no right or wrong answers.

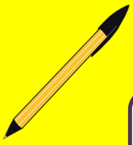

Concerns		Tips
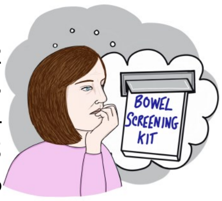 If I feel scared about bowel screening... ●		● … then I'll read the instructions. 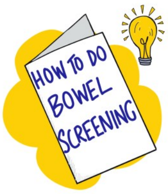
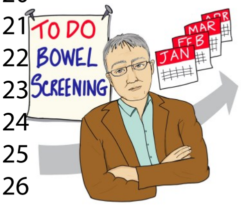 If I keep putting off using the kit... ●		● … then I'll think that this kit could help save my life. 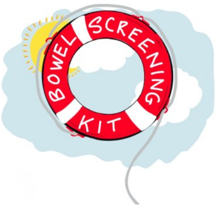
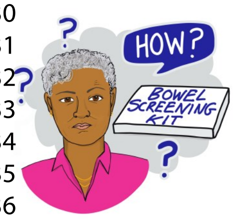 If I am not used to using a kit like this... ●		● … then I'll put the kit by the toilet to remind me. 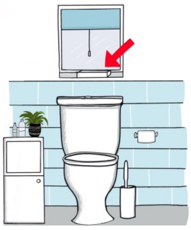
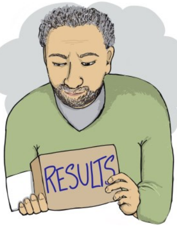 If I am worried what it might find... ●		● … then I'll wash my hands after using the kit. 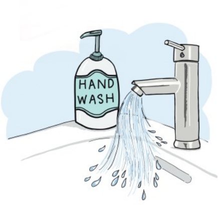
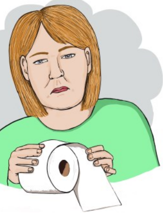 If I think using the kit is messy... ●		● ...then I'll tell myself that I'm responsible for my health. 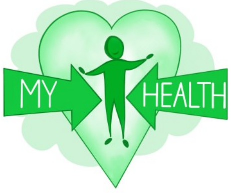
Do you have another concern? Please write it here: *If I…* ●<br>__________<br>__________<br>__________		● Got a better tip? Please write it here: ...then *I'll…*<br>__________<br>__________<br>__________

Reporting checklist for protocol of a clinical trial.

Based on the SPIRIT guidelines.

Instructions to authors

Complete this checklist by entering the page numbers from your manuscript where readers will find each of the items listed below.

Your article may not currently address all the items on the checklist. Please modify your text to include the missing information. If you are certain that an item does not apply, please write "n/a" and provide a short explanation.

Upload your completed checklist as an extra file when you submit to a journal.

In your methods section, say that you used the SPIRITreporting guidelines, and cite them as:

Chan A-W, Tetzlaff JM, Gøtzsche PC, Altman DG, Mann H, Berlin J, Dickersin K, Hróbjartsson A, Schulz KF, Parulekar WR, Krleža-Jerić K, Laupacis A, Moher D. SPIRIT 2013 Explanation and Elaboration: Guidance for protocols of clinical trials. BMJ. 2013;346:e7586

		Reporting Item	Page Number
**Administrative information**			
Title	#1	Descriptive title identifying the study design, population, interventions, and, if applicable, trial acronym	1

For peer review only

Trial registration	#2a	Trial identifier and registry name. If not yet registered, name of intended registry	2
Trial registration: data set	#2b	All items from the World Health Organization Trial Registration Data Set	n/a
Protocol version	#3	Date and version identifier	n/a
Funding	#4	Sources and types of financial, material, and other support	12
Roles and responsibilities: contributorship	#5a	Names, affiliations, and roles of protocol contributors	11
Roles and responsibilities: sponsor contact information	#5b	Name and contact information for the trial sponsor	10
Roles and responsibilities: sponsor and funder	#5c	Role of study sponsor and funders, if any, in study design; collection, management, analysis, and interpretation of data; writing of the report; and the decision to submit the report for publication, including whether they will have ultimate authority over any of these activities	12
Roles and responsibilities: committees	#5d	Composition, roles, and responsibilities of the coordinating centre, steering committee, endpoint adjudication committee, data management team, and	n/a

For peer review only

		other individuals or groups overseeing the trial, if applicable (see Item 21a for data monitoring committee)
**Introduction**		
Background and rationale	#6a	Description of research question and justification for undertaking the trial, including summary of relevant studies (published and unpublished) examining benefits and harms for each intervention
Background and rationale: choice of comparators	#6b	Explanation for choice of comparators
Objectives	#7	Specific objectives or hypotheses
Trial design	#8	Description of trial design including type of trial (eg, parallel group, crossover, factorial, single group), allocation ratio, and framework (eg, superiority, equivalence, non-inferiority, exploratory)
**Methods: Participants, interventions, and outcomes**		
Study setting	#9	Description of study settings (eg, community clinic, academic hospital) and list of countries where data will be collected. Reference to where list of study sites can be obtained

For peer review only

Eligibility criteria	#10	Inclusion and exclusion criteria for participants. If applicable, eligibility criteria for study centres and individuals who will perform the interventions (eg, surgeons, psychotherapists)
Interventions: description	#11a	Interventions for each group with sufficient detail to allow replication, including how and when they will be administered
Interventions: modifications	#11b	Criteria for discontinuing or modifying allocated interventions for a given trial participant (eg, drug dose change in response to harms, participant request, or improving / worsening disease)
Interventions: adherance	#11c	Strategies to improve adherence to intervention protocols, and any procedures for monitoring adherence (eg, drug tablet return; laboratory tests)
Interventions: concomitant care	#11d	Relevant concomitant care and interventions that are permitted or prohibited during the trial
Outcomes	#12	Primary, secondary, and other outcomes, including the specific measurement variable (eg, systolic blood pressure), analysis metric (eg, change from baseline, final value, time to event), method of aggregation (eg, median, proportion), and time point for each outcome. Explanation of the clinical relevance of chosen efficacy and harm outcomes is strongly recommended

For peer review only

Participant timeline	#13	Time schedule of enrolment, interventions (including any run-ins and washouts), assessments, and visits for participants. A schematic diagram is highly recommended (see Figure)
Sample size	#14	Estimated number of participants needed to achieve study objectives and how it was determined, including clinical and statistical assumptions supporting any sample size calculations
Recruitment	#15	Strategies for achieving adequate participant enrolment to reach target sample size
**Methods: Assignment of interventions (for controlled trials)**		
Allocation: sequence generation	#16a	Method of generating the allocation sequence (eg, computer-generated random numbers), and list of any factors for stratification. To reduce predictability of a random sequence, details of any planned restriction (eg, blocking) should be provided in a separate document that is unavailable to those who enrol participants or assign interventions
Allocation concealment mechanism	#16b	Mechanism of implementing the allocation sequence (eg, central telephone; sequentially numbered, opaque,

For peer review only

		sealed envelopes), describing any steps to conceal the sequence until interventions are assigned
Allocation: implementation	#16c	Who will generate the allocation sequence, who will enrol participants, and who will assign participants to interventions
Blinding (masking)	#17a	Who will be blinded after assignment to interventions (eg, trial participants, care providers, outcome assessors, data analysts), and how
Blinding (masking): emergency unblinding	#17b	If blinded, circumstances under which unblinding is permissible, and procedure for revealing a participant's allocated intervention during the trial
**Methods: Data collection, management, and analysis**		
Data collection plan	#18a	Plans for assessment and collection of outcome, baseline, and other trial data, including any related processes to promote data quality (eg, duplicate measurements, training of assessors) and a description of study instruments (eg, questionnaires, laboratory tests) along with their reliability and validity, if known. Reference to where data collection forms can be found, if not in the protocol

For peer review only

Data collection plan: retention	#18b	Plans to promote participant retention and complete follow-up, including list of any outcome data to be collected for participants who discontinue or deviate from intervention protocols
Data management	#19	Plans for data entry, coding, security, and storage, including any related processes to promote data quality (eg, double data entry; range checks for data values). Reference to where details of data management procedures can be found, if not in the protocol
Statistics: outcomes	#20a	Statistical methods for analysing primary and secondary outcomes. Reference to where other details of the statistical analysis plan can be found, if not in the protocol
Statistics: additional analyses	#20b	Methods for any additional analyses (eg, subgroup and adjusted analyses)
Statistics: analysis population and missing data	#20c	Definition of analysis population relating to protocol non-adherence (eg, as randomised analysis), and any statistical methods to handle missing data (eg, multiple imputation)

Methods: Monitoring

Data monitoring: formal committee	#21a	Composition of data monitoring committee (DMC); summary of its role and reporting structure; statement of whether it is independent from the sponsor and competing interests; and reference to where further

For peer review only

		details about its charter can be found, if not in the protocol. Alternatively, an explanation of why a DMC is not needed
Data monitoring: interim analysis	#21b	Description of any interim analyses and stopping guidelines, including who will have access to these interim results and make the final decision to terminate the trial
Harms	#22	Plans for collecting, assessing, reporting, and managing solicited and spontaneously reported adverse events and other unintended effects of trial interventions or trial conduct
Auditing	#23	Frequency and procedures for auditing trial conduct, if any, and whether the process will be independent from investigators and the sponsor
**Ethics and dissemination**		
Research ethics approval	#24	Plans for seeking research ethics committee / institutional review board (REC / IRB) approval
Protocol amendments	#25	Plans for communicating important protocol modifications (eg, changes to eligibility criteria, outcomes, analyses) to relevant parties (eg, investigators, REC / IRBs, trial participants, trial registries, journals, regulators)

For peer review only

Consent or assent	#26a	Who will obtain informed consent or assent from potential trial participants or authorised surrogates, and how (see Item 32)
Consent or assent: ancillary studies	#26b	Additional consent provisions for collection and use of participant data and biological specimens in ancillary studies, if applicable
Confidentiality	#27	How personal information about potential and enrolled participants will be collected, shared, and maintained in order to protect confidentiality before, during, and after the trial
Declaration of interests	#28	Financial and other competing interests for principal investigators for the overall trial and each study site
Data access	#29	Statement of who will have access to the final trial dataset, and disclosure of contractual agreements that limit such access for investigators
Ancillary and post trial care	#30	Provisions, if any, for ancillary and post-trial care, and for compensation to those who suffer harm from trial participation
Dissemination policy: trial results	#31a	Plans for investigators and sponsor to communicate trial results to participants, healthcare professionals, the public, and other relevant groups (eg, via publication, reporting in results databases, or other data sharing arrangements), including any publication restrictions

For peer review only

Dissemination policy: authorship	#31b	Authorship eligibility guidelines and any intended use of professional writers
Dissemination policy: reproducible research	#31c	Plans, if any, for granting public access to the full protocol, participant-level dataset, and statistical code

Appendices

Informed consent materials	#32	Model consent form and other related documentation given to participants and authorised surrogates
Biological specimens	#33	Plans for collection, laboratory evaluation, and storage of biological specimens for genetic or molecular analysis in the current trial and for future use in ancillary studies, if applicable

None The SPIRIT Explanation and Elaboration paper is distributed under the terms of the Creative Commons Attribution License CC-BY-NC. This checklist can be completed online using https://www.goodreports.org/, a tool made by the EQUATOR Network in collaboration with Penelope.ai

For peer review only

BMJ Open

Increasing uptake of FIT colorectal screening: protocol for the TEMPO randomised controlled trial testing a suggested deadline and a planning tool

Journal:	*BMJ Open*
Manuscript ID	bmjopen-2022-066136.R1
Article Type:	Protocol
Date Submitted by the Author:	22-Feb-2023
Complete List of Authors:	Robb, Katie; University of Glasgow<br>Kotzur, Marie; University of Glasgow, Institute of Health & Wellbeing<br>Young, Ben; University of Glasgow<br>McCowan, Colin ; University of St. Andrews<br>Hollands, Gareth; University College London<br>Irvine, Audrey; NHS Tayside<br>MacDonald, Sara; University of Glasgow<br>McConnachie, Alex; University of Glasgow<br>O'carroll, Ronan; University of Stirling<br>O'Connor, Rory; University of Glasgow, Institute of Health and Wellbeing<br>Steele, Robert; University of Dundee, Surgery and Molecular Oncology
**Primary Subject Heading**:	Public health
Secondary Subject Heading:	Oncology
Keywords:	Gastrointestinal tumours < ONCOLOGY, PUBLIC HEALTH, PREVENTIVE MEDICINE

SCHOLARONE™
Manuscripts

Increasing uptake of FIT colorectal screening: protocol for the TEMPO randomised controlled trial testing a suggested deadline and a planning tool

Kathryn A. Robb*—School of Health and Wellbeing, University Of Glasgow, Glasgow, UK

Marie Kotzur—School of Health & Wellbeing, University Of Glasgow, Glasgow, UK

Ben Young—School of Health & Wellbeing, University Of Glasgow, Glasgow, UK

Colin McCowan—School of Medicine, University Of St. Andrews, St. Andrews, UK

Gareth J. Hollands—EPPI Centre, UCL Social Research Institute, University College London, London, UK

Audrey Irvine — Scottish Bowel Screening Centre, NHS Tayside, Dundee, UK

Sara Macdonald—School of Health & Wellbeing, University Of Glasgow, Glasgow, UK

Alex McConnachie— School of Health & Wellbeing, University Of Glasgow, Glasgow, UK

Ronan E. O'Carroll—Psychology, University of Stirling, Stirling, UK

Rory C. O'Connor— School of Health & Wellbeing, University Of Glasgow, Glasgow, UK

Robert J.C. Steele—School of Medicine, University of Dundee, Dundee, UK

* corresponding author: Prof Kathryn A. Robb| School of Health and Wellbeing | Admin Building | Gartnavel Royal Hospital | 1055 Great Western Road | Glasgow G12 0XH; Katie.robb@glasgow.ac.uk; +44 (0)141 211 0685.

Word count: 4460

For peer review only

Abstract

Introduction: Screening can reduce deaths from colorectal cancer (CRC). Despite high levels of public enthusiasm, participation rates in population CRC screening programmes internationally remain persistently below target levels. Simple behavioural interventions such as completion goals and planning tools may support participation among those inclined to be screened but who fail to act on their intentions. This study aims to evaluate the impact of: a) a suggested deadline for return of the test; b) a planning tool; and c) the combination of a deadline and planning tool on return of faecal immunochemical tests (FITs) for CRC screening.

Methods and analysis: A randomised controlled trial of 40,000 adults invited to participate in the Scottish Bowel Screening Programme will assess the individual and combined impact of the interventions. Trial delivery will be integrated into the existing CRC screening process. The Scottish Bowel Screening Programme mails FITs to people aged 50-74 with brief instructions for completion and return. Participants will be randomised to one of eight groups: 1) no intervention; 2) suggested deadline (1-week); 3) suggested deadline (2-weeks); 4) suggested deadline (4-weeks); 5) planning tool; 6) planning tool plus suggested deadline (1-week); 7) planning tool plus suggested deadline (2-weeks); 8) planning tool plus suggested deadline (4-weeks). The primary outcome is return of the correctly completed FIT at three months. To understand the cognitive and behavioural mechanisms and to explore the acceptability of both interventions, we will survey (n=2000) and interview (n=40) a subgroup of trial participants.

Ethics and dissemination: The study has been approved by the National Health Service South Central—Hampshire B Research Ethics Committee (ref. 19/SC/0369). The findings will be disseminated through conference presentations and publication in peer-reviewed journals. Participants can request a summary of the results.

Trial registration The trial is registered prospectively at clinicaltrials.gov (Identifier: NCT05408169, registration date: 07.06.2022).

Article summary

Strengths and limitations of this study

1. A suggested deadline for kit return and a FIT planning tool are highly cost-effective (if effective) interventions that could be easily implemented in routine practice, through adding a sentence to the invitation letter and including a sheet of paper, respectively.
2. A 2x4 factorial RCT design will allow us to test the individual and combined impact of two behavioural interventions on colorectal screening uptake.
3. Integrating the RCT delivery into the existing colorectal screening process will provide evidence of the feasibility of providing either or both within a well-established national screening programme.
4. Cross-sectional and qualitative evaluation research will provide further insight into the psychological mechanisms that underlie any intervention effects.

For peer review only - http://bmjopen.bmj.com/site/about/guidelines.xhtml

5. While the study supports key priorities within the UK Cancer Strategies to increase uptake of FIT colorectal screening, if the interventions are effective, this will lead to an increase in demand on follow up colonoscopy which currently has limited capacity.

Keywords (3-10) colorectal cancer; screening; faecal immunochemical test; deadline; planning; randomised controlled trial; qualitative interview

For peer review only

INTRODUCTION

Colorectal cancer (CRC) is the second leading cancer killer in Scotland and worldwide.[1,2] Screening has been shown to reduce deaths from CRC if enough people invited participate.[3,4] The challenge is that high uptake is hard to achieve, and remains persistently below the target levels internationally. For example, screening rates are 44% in Australia and 62% in the USA.[5,6] Following the introduction of the single-sample faecal immunochemical test (FIT) in 2017, screening uptake in Scotland has risen to 65%, thus exceeding the Scottish target rate of 60%,[7] but remains just below the internationally accepted European Union target uptake rate of at least 66%.[8] Although this is higher than the European population-based CRC screening programmes average of 25%,[8] one third of the population still do not screen and uptake is unevenly distributed across socioeconomic factors leading to health inequalities.[7]

Theoretical rationale

CRC screening uptake remains sub-optimal, despite high levels of public enthusiasm. In a Scottish survey, 85% of people reported intending to complete the FIT,[9] yet participation is 65%.[7] This suggests that apparent enthusiasm for the new FIT is not being translated into uptake. A major reason for this disparity may be that people are 'not getting round to it',[10] reflecting the well-recognised intention-behaviour gap and consistent with the observation that much of our behaviour is determined by factors other than conscious, deliberative control.[11-14] This disparity is consistent with previous work on 'inclined abstainers',[13] showing that a proportion of people are inclined to participate in screening but fail to do it. This explanation seems particularly likely for CRC screening which, unusually for a screening test, is self-completed at home. General open-ended intentions to act often fail to lead to the desired behaviour.[15] Our previous research has found that people often put off or forget to complete and return the FIT.[16] Additional support through prompting the setting of FIT completion goals and planning may better support participation among those inclined to complete the FIT but who might otherwise fail to act.

Goal setting

Goal setting, and particularly action-planning, can help initiate behaviour in those inclined to act. This is achieved by prompting a person to specifically plan when, where and how to act, which lessens the decision-making burden when that situation is encountered.[17-19] For example, a suggested deadline by which a task should be completed can be prompt to undertaking action planning. Currently, CRC screening invitations in Scotland provide detailed information about 'where' and 'how to act' but 'when to act' is left open, potentially reducing the likelihood that recipients will plan and act accordingly. In breast and cervical screening, providing a fixed appointment time increased uptake compared to an open invitation.[20] Similarly, in our recent qualitative study, women who attended breast and cervical screening but had not completed CRC screening described how the lack of an appointment facilitated the delay and forgetting of CRC screening: "If it's a bowel screening one, yep, put it somewhere and think 'Yes, I'll do that' and then forget about it because it doesn't have an appointment date. I think if something has an appointment date, you're forced to act." (54

For peer review only - http://bmjopen.bmj.com/site/about/guidelines.xhtml

years, not screened for CRC).[16(p.6)] Recent evidence suggests that both short (1-week) and long (3-week) deadlines achieve small but significant increases in FIT return.[21] Optimal deadline length, however, has yet to be determined. It is important to establish if a suggested deadline for FIT kit return can increase CRC screening participation compared to an open invitation in a population-based screening programme.

Planning support

Systematic reviews clearly demonstrate that interventions supporting people to plan how to enact a behaviour (including action and coping planning) are particularly suited to changing the behaviour of inclined abstainers.[12] Indeed, planning support interventions have been shown to be more effective than other approaches, such as self-monitoring or providing instructions,[22] and have high adherence rates.[23] Planning interventions are often based on implementation intentions which promote behaviour change using 'if-then' plans and have successfully reduced alcohol consumption and smoking, and increased cancer screening attendance and physical activity.[11,12,24,25] Previous research on implementation intention interventions for CRC screening has had limited success when it has relied on prepared statements of barriers ('if') and possible solutions ('then').[26] Encouraging people to form their own implementation intentions for CRC screening appears to produce clinically significant increases in uptake.[24] Planning tools (also called volitional help sheets [VHS]) emphasise this by requiring users to physically draw a line to link a barrier e.g. "If I am tempted to eat when I am anxious" with a solution e.g. "then I will reward myself when I do not overeat." to ensure user-engagement.[27(p.706)] The rationale is that the 'if' barriers or critical situations are made personalised and salient and, as a result, the 'then' response or solution comes automatically to mind.

The Medical Research Council's recently updated guidance on complex intervention development and evaluation recommends addressing questions beyond intervention effectiveness, such as "whether and how the intervention will be acceptable, implementable, cost effective, scalable, and transferable across contexts.".[28(p.2)] With a view to implementing a suggested deadline or planning tool at national scale, if effective, the acceptability of both interventions to CRC screening service users must be investigated.

Objectives

The overall aim of the TimE fraMe and Planning tOol (TEMPO) study is to evaluate the impact of a suggested deadline and planning tool on FIT return in the Scottish Bowel Screening Programme.

The study addresses six research questions:

1. Does providing a suggested deadline increase uptake of CRC screening?
2. Does providing a FIT planning tool increase uptake of CRC screening?
3. Does providing both a suggested deadline and a FIT planning tool increase uptake of CRC screening?
4. Does the length of the suggested deadline (one, two or four weeks) impact upon uptake of CRC screening?

For peer review only - http://bmjopen.bmj.com/site/about/guidelines.xhtml

5. What are the cognitive and behavioural mechanisms underlying any observed effect of providing a suggested deadline, a planning tool, or a combination of both?
6. How acceptable do participants find receiving a suggested deadline, a planning tool, or a combination of both?

METHODS AND ANALYSIS

The study employs a 2x4 factorial randomised controlled trial (RCT) design combined with cross-sectional quantitative and qualitative evaluation research with a sub-sample of trial participants. Figure 1 shows the CONSORT flow chart.

Study setting

This is a single-centre trial to be conducted in collaboration with the Scottish Bowel Screening Centre based in Dundee, Scotland, and integrated into the national CRC screening programme. In Scotland, colorectal screening is offered to people aged 50-74, every two years, by single-sample faecal immunochemical test (FIT).

Eligibility criteria

All people eligible for CRC screening in Scotland who have a Community Health Index Number can be included in this study.

Recruitment and allocation

Following established recruitment procedures,[29,30] working in partnership with the Scottish Bowel Screening Centre staff, 40,000 consecutive adults due to be sent a FIT will be randomised to one of eight groups (Figure 1) from 20.06.2022 over approximately 10 days.

The randomisation schedule will be computer-generated at the Robertson Centre for Biostatistics, University of Glasgow. The schedule will be stored in a secure area of the Robertson Centre for Biostatistics network, with no access for those developing the statistical analysis programs and transferred via a secure web portal to the Scottish Bowel Screening Centre team, who identify when people are to be invited for CRC screening. The IT system electronically generates the appropriate mailing packages to be sent out to each person. For this study, randomisation will be performed by the method of randomised permuted blocks of length 8, so that in every 8 packages sent, exactly one of each type will be included.

Ethical approval

Ethical approval was granted by the UK National Health Service South Central – Hampshire B Research Ethics Committee (REC ref. no. 19/SC/0369). Written informed consent will not be obtained before inclusion in the trial to maintain ecological validity and to avoid selection bias. This is a common approach in comparable trials performed in the context of national screening programmes, [29, 30] without which it may not be viable to perform robust evaluations leading to evidence-based service improvements. There is very low risk of the interventions causing harm to participants and individuals will not be identifiable from trial data. Consent for the questionnaire survey will be implied through the completion of the

For peer review only - http://bmjopen.bmj.com/site/about/guidelines.xhtml

questionnaire. Written informed consent to participate in a qualitative interview will be taken by the interviewer (Supplementary file 1). The research will be carried out in accordance with the World Medical Association Declaration of Helsinki (1964) and its revisions (Tokyo [1975], Venice [1983], Hong Kong [1989], South Africa [1996] and Edinburgh[2000]).

Blinding

The process of generating and mailing the routine screening invitation letters is fully automated and is conducted by a large mail-handling company. A data file containing contact details for the screening invitation letters is sent to the mail-handling company daily. The company will print screening invitation letters with suggested deadlines and add the planning tool in accordance with the participants' allocation within this study. The researchers, including those performing the analysis, will therefore be blind to the allocation of participants. The participants will not be informed of the experiment.

Interventions

There will be eight allocation groups (Figure 1): Group 1 – standard invitation (n = 5,000); Group 2 – 1-week deadline (n=5,000); Group 3 – 2-week deadline (n=5,000); Group 4 – 4-week deadline (n=5,000); Group 5 – planning tool (n=5,000); Group 6 – planning tool plus 1-week deadline (n=5,000); Group 7 – planning tool plus 2-week deadline (n=5,000); Group 8 – planning tool plus 4-week deadline (n=5,000).

Deadline intervention

Those allocated to the deadline intervention (groups 2-4 and 6-8) will receive a screening invitation letter with one of three suggested deadlines added (Table 1): 1 week, 2 weeks and 4 weeks. This choice was based on feedback from the Cancer Research UK Early Diagnosis Advisory Group, public and patient involvement representatives, and observation of kit return patterns in the Scottish Bowel Screening Programme. The deadline will be displayed centrally on the standard CRC screening invitation letter as a bold, highlighted sentence (Table 1). The wording *"or as soon as possible"* is intended to make clear that the deadline constitutes a suggested return date only, to avoid discouraging participants who are unable to meet the deadline. The existing invitation letter already states "*post it as soon as possible*" and the suggested deadline builds on this by providing a more explicit and specific goal. Furthermore, a standard reminder will be sent after 6 weeks without a deadline and so will provide a safety net for any potential participants who have missed the deadline in their first invitation letter.

Table 1 Deadline intervention arms

Intervention group	Suggested FIT return deadline	Deadline sentence
Groups 2 and 6	1-week deadline	Please return your kit within 1 week (by [insert date 10 days from posting date]) or as soon as possible.
Groups 3 and 7	2-week deadline	Please return your kit within 2 weeks (by [insert date 17

For peer review only - http://bmjopen.bmj.com/site/about/guidelines.xhtml

		days from posting date]) or as soon as possible.
Groups 4 and 8	4-week deadline	Please return your kit within 4 weeks (by [insert date 31 days from posting date]) or as soon as possible.

Planning tool intervention

Those allocated to the planning tool intervention (groups 5-8) will receive a planning tool with their screening invitation. The planning tool will be presented on a separate single sheet of paper inserted into the CRC screening invitation envelope. It asks participants to identify "*concerns*" (i.e. barriers) they have with using the FIT from a list and to link them to a "*tip*" (i.e. solution) from an adjacent list to help them overcome this concern. The planning tool reads, *"Here are some tips that people find helpful to make the bowel screening kit even easier. Try drawing a line from any concern you have to a tip which might help you. You can draw as many or as few lines as you like. There are no right or wrong answers."* Concerns reflect critical situations in conventional planning tool designs and tips reflect solutions.[27] Each concern and tip is accompanied by an image illustrating its meaning. Finally, participants will have the option to write their own concern or tip. While the planning tool instructions encourage people to use the planning tool, they do not suggest that participants must do so in order to take part in CRC screening. The specific listed concerns and tips are based on extensive development work involving consideration of existing theoretical and empirical literature, in-depth qualitative interviews, a survey of people who had been invited to complete a FIT, and co-design workshops to create a user-friendly planning tool with members of the public who have completed the FIT and others who have not completed the FIT.[31-33] The final planning tool was reviewed and approved by the Research Team, Patient and Public Involvement representatives, and a patient and public group of the Scottish Primary Care Network.

Table 2 displays the concerns and tips shown in the planning tool. The planning tool is presented in Supplementary file 2.

Table 2 Planning tool intervention—concerns and tips

Concerns	Tips
If I feel scared about bowel screening…	… then I'll read the instructions.
If I keep putting off using the kit…	… then I'll think this this kit could help save my life.
If I am not used to using a kit like this…	… then I'll put the kit by the toilet to remind me.
If I am worried what it might find…	… then I'll wash my hands after using the kit.
If I think using the kit is messy…	… then I'll tell myself that I'm responsible for my health.

Control group

As the trial has a factorial design, it follows that the control group for the assessment of the independent effect of each intervention is all those who did not receive the intervention. This is because any impact of one intervention impacts to an equal extent those who do and do

For peer review only - http://bmjopen.bmj.com/site/about/guidelines.xhtml

not receive the other intervention. Therefore, groups 1 and 5 together make up the control group for the assessment of suggested deadlines and groups 1-4 make up the control group for assessment of the planning tool. Only participants in Group 1 will be sent a standard CRC screening invitation and FIT (without a suggested deadline or a planning tool) according to normal current practice. It is also important to note that three groups will receive both a planning tool and a suggested deadline, which will allow assessments of the combined effects of a suggested deadline and the planning tool compared to receiving a deadline only, a planning tool only, or neither.

Evaluation research of cognitive and behavioural mechanisms and acceptability

We will conduct two studies answering research questions 5 and 6.

Questionnaire survey

We will conduct a questionnaire-based case-control study to assess the cognitive and behavioural mechanisms underlying any effect of providing a suggested deadline for FIT kit return and a planning tool.

Recruitment. At least three months after the screening invitation, a subsample of trial participants will be identified from all eight groups, including people who returned (case) and did not return (control) their FIT, and people living in 40% most and least deprived neighbourhoods (based on SIMD quintiles). The subsample will be sent a postal invitation to complete and return a survey. The aim is to obtain responses from approximately 1,000 people who have returned the FIT (case) and 1,000 who have not returned (control) the FIT, across the eight groups. Based on previous work, we anticipate achieving a higher response rate from those returning the FIT and a lower response from those not returning the FIT, therefore approximately 11,872 questionnaires will be mailed, as shown in Figure 1.[32] A reminder questionnaire will be sent to those who have not returned the questionnaire after three weeks.

Materials and procedure. A brief questionnaire will include items to assess cognitive and behavioural mechanisms, e.g. action and coping planning,[17] that were previously adapted for the CRC screening context. [19, 32] It will also include a theory-informed acceptability measure that has been subject to a pre-validation method but does not yet have fully established psychometric properties,[34] as well as demographic characteristics. Participants will be asked if they are willing to participate in future research to assist recruitment for qualitative interviews.

Qualitative interviews

We will conduct a qualitative interview study, adopting a phenomenological approach to explore participants' experiences of receiving a FIT with and without the interventions, and explore acceptability of the interventions.

Recruitment. Interview participants will be sampled from survey participants who have indicated willingness to participate in further research. We will recruit approximately 40 participants and will aim to achieve a balance of participants who have and have not returned the FIT, men and women, from across the CRC screening invitation age range (50-74 years),

from both deprived and affluent neighbourhoods (based on SIMD), and from the trial intervention groups. The estimated sample size is based on the principles of achieving data saturation and what is pragmatic, yet informative.

Procedure. Potential participants will be contacted to request participation in a semi-structured qualitative interview conducted face-to-face, by telephone or video-call. Interviews will last approximately 30-60 minutes, and topic guides (Supplementary file 3) will be used. Participants will be offered £30 towards travel and expenses.

Outcomes

Our primary outcome is the proportion of FITs returned correctly completed to be tested by the CRC screening laboratory providing a positive or negative result, within three months of the FIT being sent to a person. The return and successful testing of any replacement FITs that were sent to people after the first FIT at study baseline will be treated as a FIT return. As secondary outcomes, we will evaluate cognitive and behavioural mechanisms and explore the acceptability of both interventions.

Sample size

This 2x4 factorial RCT involves the assessment of two interventions, so each will be assessed at a significance level of 2.5%, to preserve an overall Type I error rate of 5%.

The assessment of the effect of the planning tool will involve the comparison of two groups of 20,000 participants. At a significance level of 2.5%, this gives 90% power to detect an increase in uptake rates from 65% to 66.8%. Alternatively, if the intervention increases uptake rates from 65% to 68%, the 97.5% confidence interval for the difference in uptake rates will have a width of ±1.06%.

The assessment of the effect of suggested deadlines for FIT kit return will involve four groups of 10,000 participants. Comparing each suggested deadline with the control group (no deadline) at a significance level of 0.83% (one third of 2.5%), there will be 90% power to detect increases in uptake rates from 65% to 67.63%. If an intervention increases uptake from 65% to 68%, the 99.17% confidence interval for the difference in uptake rates will have a width of ±1.76%.

Survey. The study is a case-control study (1,000 cases, 1,000 controls). The study will have 90% power at a 5% significance level to detect an odds ratio for being a case of 1.146 per standard deviation increase in a continuous predictor variable; for a binary predictor with 50% prevalence, the study will have 90% power to detect an odds ratio for being a case of 1.345; for a binary predictor with a prevalence of 10%, the study will have 90% power to detect an odds ratio for being a case of 1.662.

Trial data collection and management

For peer review only

For the purposes of this study, two additional variables will be added to the Scottish Bowel Screening Programme invitation data file: i) a unique identifier for each participant; and ii) a variable coding for the allocated condition. The pseudonymised participant data including FIT kit return data will be transferred from the Scottish Bowel Screening Centre in Dundee to the Robertson Centre for Biostatistics at the University of Glasgow using a secure file transfer protocol.

Data analysis plan

Primary and secondary outcome measures will be summarised using descriptive statistics.

Primary outcome

The primary analysis will use a logistic regression model, including a binary variable for whether the planning tool was provided, and a four-level categorical variable denoting which deadline was suggested. Age, sex, and area deprivation (measured by the Scottish Index of Multiple Deprivation [SIMD][35]) will be included in the model, to improve model specification. The main effects of each intervention will be reported as an odds ratio, with a confidence interval (CI) and p-value. For the estimate of the effect of the planning tool, a 97.5% CI will be reported. For estimates of the effect of alternative suggested deadlines, relative to no deadline, 99.17% CIs will be used. Subsequent analyses will model the interaction between the two interventions, to assess whether the interventions have independent effects or are synergistic. We shall also model interactions between each intervention effect and age, sex, and deprivation, to assess whether the interventions have different effects for subgroups of the population. Assessing uptake by deprivation is important given the disparities in screening uptake by deprivation (53% most deprived versus 73% least deprived;[7]) which any intervention could reduce or exacerbate.[36]

Survey analysis. Descriptive statistics, Chi-square tests, ANOVA and logistic regression will be used to examine cognitive and behavioural mechanism differences between the intervention and control groups and their association with FIT kit return.

Qualitative analysis. The interviews will be audio-recorded, transcribed verbatim, checked by the interviewer and analysed using the Framework Method.[37] This method facilitates systematic, rigorous and transparent data management, providing an audit trail from raw data to final themes. We will use NVivo software to undertake coding, manage and apply a working analytical framework, generate framework matrices and summarise and interpret data. This method will adopt a combined deductive and inductive approach, guided by the behavioural mechanisms targeted by the interventions (e.g. action and coping planning),[17] as well as constructs within the theoretical framework of acceptability,[38] whilst also allowing other experiences and any unintended intervention effects to be explored. BY will lead the analysis with support from KAR, MK and SM. Interpretation of findings may also involve the wider co-investigator group, including the project Patient & Public Representatives, for improved credibility of findings. In addition, the results from the survey will be used to inform the analysis of the qualitative interviews and vice versa to determine whether synthesising the two data sources can provide unique insights which would not be apparent by analysing the data independently. Calls to the Scottish Bowel Screening

For peer review only - http://bmjopen.bmj.com/site/about/guidelines.xhtml

Programme helpline relating to the suggested deadline for FIT kit return and planning tool will also be monitored via a call report form including date, time and nature of query as an additional assessment of acceptability.

Participant timeline

This protocol describes a three-month project as shown in Figure 2.

Study management and monitoring

This trial combines two individually funded studies with the same Principal Investigator. The trial will be managed by the co-investigator teams of both studies including the Principal Investigator, academic co-investigators, key researchers, and public and patient involvement representatives who will oversee trial conduct and progress together. Data management, participant confidentiality and the conduct of all trial staff will adhere to the protocol (Version 1.4, 06.09.2021 or subsequent approved versions), Good Clinical Practice guidelines, and the Data Protection Act 2018. An audit trail of all documentation and data collection will be kept in an electronic study site file to facilitate monitoring by the research team and the sponsor. This trial is sponsored by the NHS Greater Glasgow and Clyde. Amendments to the protocol will be notified to the sponsor and the responsible Research Ethics Committee for their approval. The sponsor may audit the trial at any time and without notice.

Stopping criteria

The CRC screening invitations and kits will be posted as one batch over approximately 10 days. There are no stopping criteria for recruitment.

Harms

This is a low-risk trial providing people eligible for the Scottish Bowel Screening Programme with additional written information. While we recognise that providing a suggested deadline or a planning tool may increase the burden of completing the FIT, we will ensure that participants: a) are not disadvantaged if they cannot meet the suggested deadline for returning the FIT; and b) are under no obligation to use the planning tool in order to complete CRC screening. We will be able to evaluate the additional burden of each intervention in the questionnaire and interview evaluation studies.

Public and patient involvement

The study is supported by two patient and public representatives (PPRs) who have provided input throughout the programme of research. The PPRs were involved in the design of the study, the creation of materials to support the recruitment and join the co-investigator meetings to discuss study progress. At the end of the study, the PPRs will review a plain language summary of the study findings which we will make available to the interview participants.

Intervention materials for this study, i.e. the planning tool and the screening invitation letters including the three suggested deadlines, have been developed in workshops and focus groups and have received feedback from the PPRs, the Cancer Research UK Early Diagnosis Advisory Group, and the PPRs of the Scottish Primary Care Research network.

DISCUSSION

For peer review only - http://bmjopen.bmj.com/site/about/guidelines.xhtml

The purpose of this study is to increase uptake of FIT colorectal screening using two behavioural interventions. The study will evaluate the impact of a suggested deadline, a planning tool, and the combination of a deadline and a planning tool on return of FITs. This research is important because colorectal cancer is the second most common cause of cancer death globally [1] but uptake remains below target levels.

The study builds on existing theoretical and empirical evidence [11-17; 27] and is the first attempt to test the individual and combined impact of a deadline and a co-designed planning tool within the real-world setting of a national screening programme. In addition, this study will provide evidence on the most effective timeframe for a suggested deadline as well as the cognitive and behavioural mechanisms that underlie any intervention effects. Finally, the study will investigate the acceptability of providing either intervention to those invited to participate in CRC screening. If effective, the interventions will be highly cost effective. A simple change to the wording of the colorectal screening programmes' invitation letters (deadline) and the inclusion of a sheet of paper (planning tool) will be all that is required to immediately see the benefits of increased uptake and subsequent earlier diagnosis of colorectal cancer.

The study has a number of potential limitations. While the study supports key priorities within the UK Cancer Strategies to increase uptake of FIT colorectal screening, if the interventions are effective, this will lead to an increase in demand on follow up colonoscopy which currently has limited capacity. The primary outcome is return of a kit after 3 months rather than 6 months as used by the Scottish Bowel Screening Programme. This was because the study was severely delayed by the COVID-19 pandemic and the follow up was consequently reduced. It is unclear whether the results would be generalisable to screening programmes beyond the UK. The secondary outcomes examining the cognitive and behavioural mechanisms underlying any observed effects and the acceptability of the interventions will only be assessed after three months. Participants may not recall in detail their experience of receiving the deadline and/or the planning tool. Assessing the potential mechanisms immediately before and after test completion my have provided greater insight but this design risks contaminating the intervention itself. Our retrospective approach therefore provides the most appropriate option in this applied setting.

DISSEMINATION

The results from this study will be presented at national and international conferences and written up for publication in peer reviewed journals. The study is expected to produce two manuscripts: i) primary findings of the RCT and ii) the findings of the secondary evaluation. The co-authors have a track record of public and media engagement in research. They will work closely with the University Of Glasgow Press Office to disseminate the findings to the public. Study participants will receive a summary of the findings if they request this.

DECLARATIONS

For peer review only - http://bmjopen.bmj.com/site/about/guidelines.xhtml

Acknowledgements
We thank the PPRs, Mary Cameron and Lucy J. Robertson, for their continued support of this research, the patient and public involvement group of the Scottish Primary Care Research Network for their feedback on the intervention materials, and Catherin McKay of ListenThinkDraw who provided illustrative services during the development of the planning tool and images for the final version.

Author contributions
KAR conceived both the planning tool and deadline interventions. KAR and MK designed the intervention procedures, study materials and evaluation with input from BY, SM, REOC, RCOC, AI, CMC, AM, GJH and RJCS. AM conducted the sample size calculations and designed the statistical analyses. KAR submitted the original application for ethical approval and MK and BY drafted and managed approval amendments. KAR, BY and MK drafted the protocol manuscript. All co-authors read and approved the final manuscript.

Funding
This study is supported by an Early Diagnosis Advisory Group (EDAG) Project Grant from Cancer Research UK (C9227/A27877 - Increasing uptake of faecal immunochemical test (FIT) bowel screening: trial of providing a suggested deadline for FIT kit return, PI KAR, Co-investigators: CMC, AM, GJH, RJCS) and by a Response Mode Grant from the Scottish Chief Scientist Office (HIPS/17/23 - Increasing uptake of bowel cancer screening: development of a FIT planning support tool, PI: KAR, Co-investigators: SM, REOC, RCOC, AI, RJCS, MK). The funders have no involvement in the study design, data collection or analysis, or the writing and publication of reports of the study. All researchers involved in this study were independent of the funder.

Competing interests
The authors declare that they have no competing interests.

Consent for publication
Not applicable

Availability of data and materials
Not applicable

For peer review only

REFERENCES

1. Ferlay J, Colombet M, Soerjomataram I, Parkin DM, Piñeros M, Znaor A, Bray F. Cancer statistics for the year 2020: An overview. Int J Cancer. 2021;149:778-789.
2. Public Health Scotland. Cancer Mortality in Scotland: Annual update to 2021. [Internet]. 2022. Available from https://publichealthscotland.scot/media/15989/2022-10-25-cancer-mortality-report.pdf
3. Lin JS, Perdue LA, Henrikson NB, Bean SI, Blasi PR. Screening for Colorectal Cancer: Updated Evidence Report and Systematic Review for the US Preventive Services Task Force. JAMA. 2021;325(19):1978–1998.
4. Randel KR, Schult AL, Botteri E, Hoff G, Bretthauer M, Ursin G, Natvig E, Berstad P, Jørgensen A, Sandvei PK, Olsen ME, Frigstad SO, Darre-Næss O, Norvard ER, Bolstad N, Kørner H, Wibe A, Wensaas KA, de Lange T, Holme Ø. Colorectal Cancer Screening With Repeated Fecal Immunochemical Test Versus Sigmoidoscopy: Baseline Results From a Randomized Trial. Gastroenterology. 2021 Mar;160(4):1085-1096.e5.
5. Australian Institute of Health and Welfare National Bowel Cancer Screening Program monitoring report 2022. 2022; catalogue number CAN 148, AIHW, Australian Government.
6. White A, Thompson TD, White MC, et al. Cancer screening test use—United States, 2015. *MMWR Morb Mortal Wkly Rep* 2017;66(8):201.
7. Public Health Scotland. Scottish Bowel Screening Programme Statistics: For the period of invitations between May 2019 and April 2021. [Internet]. 2022. [cited 2022 June 23] Available at https://publichealthscotland.scot/media/11605/2022-02-08-bowel-screening-publication-report.pdf.
8. Senore C, Basu P, Anttila A, et al. Performance of colorectal cancer screening in the European Union Member States: data from the second European screening report. *Gut* 2019;68(7):1232-44.
9. Chambers JA, Callander AS, Grangeret R, et al. Attitudes towards the Faecal Occult Blood Test (FOBT) versus the Faecal Immunochemical Test (FIT) for colorectal cancer screening: Perceived ease of completion and disgust. *BMC Cancer* 2016;16(1):96-102.
10. Lo SH, Waller J, Wardle J, et al. Comparing barriers to colorectal cancer screening with barriers to breast and cervical screening: A population–based survey of screening-age women in Great Britain. *J Med Screen* 2013;20(2):73-79.
11. Gollwitzer PM, Sheeran P. Implementation intentions and goal achievement: A meta-analysis of effects and processes. In: Advances in experimental social psychology, Vol. 38. San Diego: Elsevier Academic Press Inc. 2006:69-119.
12. Kwasnicka D, Presseau J, White M, et al. Does planning how to cope with anticipated barriers facilitate health-related behaviour change? A systematic review. *Health Psychol Rev* 2013;7(2):129-145.
13. Orbell S, Sheeran P. 'Inclined abstainers': A problem for predicting health-related behaviour. Br J Soc Psychol 1998;37(2):151-165.
14. Hollands GJ, Marteau TM, Fletcher PC. Non-conscious processes in changing health-related behaviour: A conceptual analysis and framework. *Health Psychol Rev* 2016;10(4):381–394.

For peer review only - http://bmjopen.bmj.com/site/about/guidelines.xhtml

15. Webb T, Sheeran P. Does changing behavioural intentions engender behaviour change: A meta-analysis of the experimental evidence. *Psychol Bull 2006;132:*249-268.
16. Kotzur M, McCowan C, Macdonald S, et al. Why colorectal screening fails to achieve the uptake rates of breast and cervical cancer screening: a comparative qualitative study. *BMJ Qua Saf* 2020;29(6):482-90.
17. Gollwitzer PM. Implementation intentions: Strong effects of simple plans. *Am Psychol* 1999;54(7): 493.
18. Michie S, Dormandy E, Marteau TM. Increasing screening uptake amongst those intending to be screened: the use of action plans. Patient Educ Couns 2004;55(2):218-22.
19. Presseau J, Johnston M, Heponiemi T, et al. Reflective and automatic processes in health care professional behaviour: a dual process model tested across multiple behaviours. *Ann Behav Med* 2014;48(3), 347-358.
20. Duffy SW, Myles JP, Maroni R, et al. Rapid review of evaluation of interventions to improve participation in cancer screening services. *J Med Screen* 2017;24(3): 127-145.
21. Lieberman, A., Gneezy A, Berry E. The effect of deadlines on cancer screening completion: a randomized controlled trial. *Sci Rep* 2021;11(1):13876.
22. Dombrowski SU, Sniehotta FF, Avenell A, et al. Identifying active ingredients in complex behavioural interventions for obese adults with obesity-related co-morbidities or additional risk factors for co-morbidities: A systematic review. *Health Psychol Rev* 2012;6(1):7-32.
23. O'Connor RC, Ferguson E, Scott F, et al. A randomised controlled trial of a brief psychological intervention to reduce repetition of self-harm in patients admitted to hospital following a suicide attempt. *Lancet Psychiatry* 2017;4(6):451-60.
24. Neter E, Stein N, Barnett-Griness O, et al. From the Bench to Public Health: Population-level Implementation Intentions in Colorectal Cancer Screening. *Am J Prev Med* 2014;46(3): 273-280.
25. Rutter DR, Steadman L, Quine L. An implementation intentions intervention to increase uptake of mammography. *Ann Behav Med* 2006;32(2):127-34.
26. Lo SH, Good A, Sheeran P, et al. Preformulated implementation intentions to promote colorectal cancer screening: A cluster-randomized trial. *Health Psychol* 2014;33(9):998-1002.
27. Armitage CJ, Norman P, Noor M, et al. Evidence that a very brief psychological intervention boosts weight loss in a weight loss program. *Behav Ther* 2014;45(5):700–707.
28. Skivington K, Matthews L, Simpson SA, et al. A new framework for developing and evaluating complex interventions: Update of Medical Research Council guidance. *BMJ* 2021;374.
29. Libby, G, Bray, J, Champion, J, et al. Pre-notification increases uptake of colorectal cancer screening in all demographic groups: a randomized controlled trial. *J Med Screen* 2011;8,24-29.
30. O'Carroll RE, Chambers JA, Brownlee L, et al. Anticipated regret to increase uptake of colorectal cancer screening (ARTICS): A randomised controlled trial. *Soc Sci Med.* 2015;142:118-127.

For peer review only

31. Kotzur M, Macdonald S, O'Connor R, et al. Identifying barriers and solutions to self-completed FIT bowel screening to develop a volitional help-sheet. 33rd Annual Conference of the European Health Psychology Society; 2019 September 03-07; Dubrovnik, Croatia.
32. Kotzur M, Macdonald S, O'Carroll RE, et al. What are common barriers and helpful solutions to colorectal cancer screening? A cross-sectional survey to develop intervention content for a planning support tool; BMJ Open 2022;12:e062738. .
33. Robb KA. Achieving solutions to inequalities in cancer screening. Cancer Research UK/UK Society for Behavioural Medicine Cancer Special Interest Group Behavioural Science Webinar; 2022 June 07; virtual seminar.
34. Sekhon, M., Cartwright, M. & Francis, J.J. Development of a theory-informed questionnaire to assess the acceptability of healthcare interventions. BMC Health Serv Res 22, 279 (2022).
35. Scottish Government. Scottish Index of Multiple Deprivation 2020. [Internet]. [cited 2022 May 25]. Available from https://www.gov.scot/collections/scottish-index-of-multiple-deprivation-2020/
36. Lorenc T, Petticrew M, Welch V, et al. What types of interventions generate inequalities? Evidence from systematic reviews. *J Epidemiol Community Health* 2013;67:190-193.
37. Ritchie J, Spencer L. Qualitative data analysis for applied policy research. In Ritchie J, Spencer L, Bryman A et al. eds. Analysing Qualitative Data. London, UK: Routledge 1994:173-194.
38. Sekhon M, Cartwright M, Francis JJ. Acceptability of healthcare interventions: an overview of reviews and development of a theoretical framework. *BMC Health Serv Res* 2017;17(1):88.

For peer review only - http://bmjopen.bmj.com/site/about/guidelines.xhtml

FIGURE TITLES AND LEGENDS

Figure 1 CONSORT flow chart

Figure 2 Participant timeline
Note. * denotes data collection end points

For peer review only

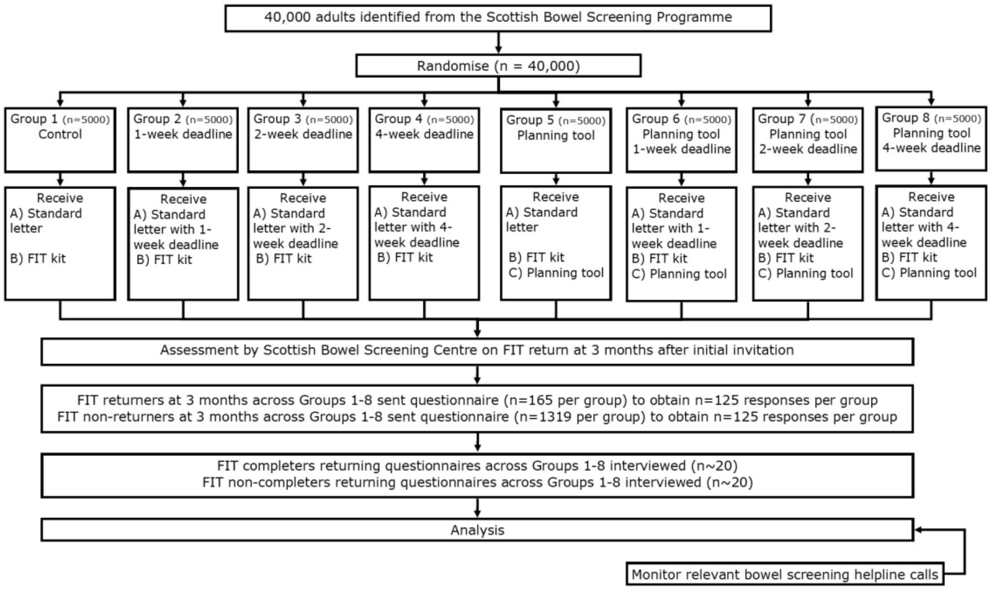

Figure 1 CONSORT flow chart

297x209mm (150 x 150 DPI)

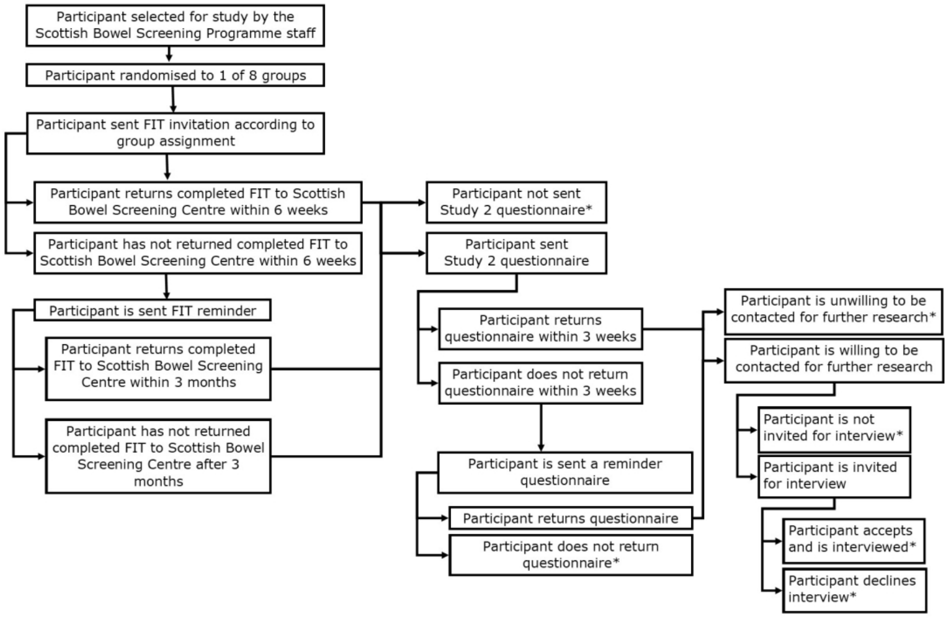

Figure 2 Participant timeline

297x209mm (150 x 150 DPI)

University of Glasgow
School of Health & Wellbeing

NHS SCOTLAND

Mental Health and Wellbeing, Gartnavel Royal Hospital, Administration Building, 1st Floor, 1055 Great Western Road, Glasgow, G12 0XH

CONSENT FORM

Title of project: **People's views on bowel screening – interview study**

Name of Researchers: Dr Ben Young and Prof Katie Robb

Please initial box

1. I confirm that I have read and understand the information sheet Version 1.3 dated 5.8.2022 for the above study. ☐
2. I have had the opportunity to consider the information, ask questions and have had these answered satisfactorily ☐
3. I agree to the interview being audio-recorded and to its contents being used for research purposes. ☐
4. I understand that my participation is voluntary and that I am free to withdraw at any time, without giving any reason, without my medical care or legal rights being affected. ☐
5. I understand that my medical notes and data collected from the study may be looked at by regulatory authorities or by persons from NHS Scotland where it is relevant to my taking part in this study. I agree to these persons having access to this information. ☐
6. I agree to take part in the above study. ☐

Name of participant	Date	Signature
Researcher	Date	Signature

1 original for participant, 1 original for researcher

For peer review only - http://bmjopen.bmj.com/site/about/guidelines.xhtml

TIPS FOR USING YOUR KIT

Here are some tips that people find helpful to make the bowel screening kit even easier. Try drawing a line from any concern you have to a tip which might help you. You can draw as many or as few lines as you like. There are no right or wrong answers.

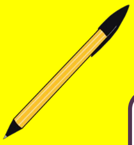

Concerns

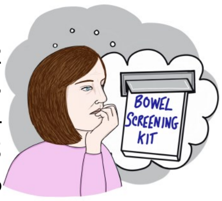

If I feel scared about bowel screening...

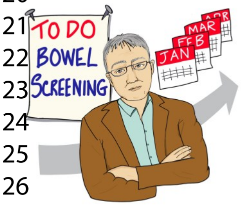

If I keep putting off using the kit...

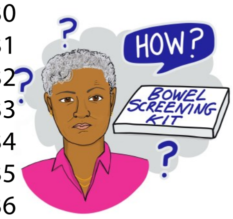

If I am not used to using a kit like this...

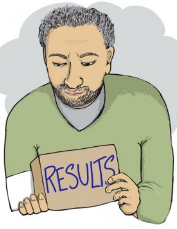

If I am worried what it might find...

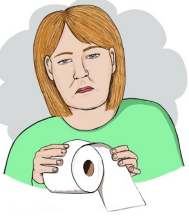

If I think using the kit is messy...

Do you have another concern? Please write it here: *If I…*

____

____

____

Tips

… then I'll read the instructions.

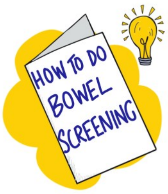

… then I'll think that this kit could help save my life.

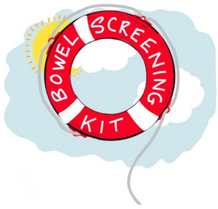

… then I'll put the kit by the toilet to remind me.

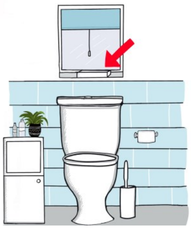

… then I'll wash my hands after using the kit.

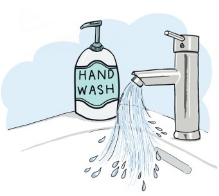

...then I'll tell myself that I'm responsible for my health.

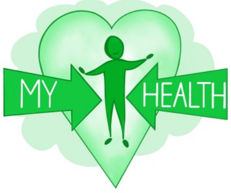

Got a better tip? Please write it here: ...then *I'll…*

____

____

____

For peer review only - http://bmjopen.bmj.com/site/about/guidelines.xhtml

Reporting checklist for protocol of a clinical trial.

Based on the SPIRIT guidelines.

Instructions to authors

Complete this checklist by entering the page numbers from your manuscript where readers will find each of the items listed below.

Your article may not currently address all the items on the checklist. Please modify your text to include the missing information. If you are certain that an item does not apply, please write "n/a" and provide a short explanation.

Upload your completed checklist as an extra file when you submit to a journal.

In your methods section, say that you used the SPIRITreporting guidelines, and cite them as:

Chan A-W, Tetzlaff JM, Gøtzsche PC, Altman DG, Mann H, Berlin J, Dickersin K, Hróbjartsson A, Schulz KF, Parulekar WR, Krleža-Jerić K, Laupacis A, Moher D. SPIRIT 2013 Explanation and Elaboration: Guidance for protocols of clinical trials. BMJ. 2013;346:e7586

		Reporting Item	Page Number
**Administrative information**			
Title	#1	Descriptive title identifying the study design, population, interventions, and, if applicable, trial acronym	1

For peer review only

Trial registration	#2a	Trial identifier and registry name. If not yet registered, name of intended registry	2
Trial registration: data set	#2b	All items from the World Health Organization Trial Registration Data Set	n/a
Protocol version	#3	Date and version identifier	n/a
Funding	#4	Sources and types of financial, material, and other support	14
Roles and responsibilities: contributorship	#5a	Names, affiliations, and roles of protocol contributors	14
Roles and responsibilities: sponsor contact information	#5b	Name and contact information for the trial sponsor	12
Roles and responsibilities: sponsor and funder	#5c	Role of study sponsor and funders, if any, in study design; collection, management, analysis, and interpretation of data; writing of the report; and the decision to submit the report for publication, including whether they will have ultimate authority over any of these activities	14
Roles and responsibilities: committees	#5d	Composition, roles, and responsibilities of the coordinating centre, steering committee, endpoint adjudication committee, data management team, and	n/a

For peer review only

		other individuals or groups overseeing the trial, if applicable (see Item 21a for data monitoring committee)	
**Introduction**			
Background and rationale	#6a	Description of research question and justification for undertaking the trial, including summary of relevant studies (published and unpublished) examining benefits and harms for each intervention	4
Background and rationale: choice of comparators	#6b	Explanation for choice of comparators	8
Objectives	#7	Specific objectives or hypotheses	5
Trial design	#8	Description of trial design including type of trial (eg, parallel group, crossover, factorial, single group), allocation ratio, and framework (eg, superiority, equivalence, non-inferiority, exploratory)	6
**Methods: Participants, interventions, and outcomes**			
Study setting	#9	Description of study settings (eg, community clinic, academic hospital) and list of countries where data will be collected. Reference to where list of study sites can be obtained	6

For peer review only

Eligibility criteria	#10	Inclusion and exclusion criteria for participants. If applicable, eligibility criteria for study centres and individuals who will perform the interventions (eg, surgeons, psychotherapists)	6
Interventions: description	#11a	Interventions for each group with sufficient detail to allow replication, including how and when they will be administered	7
Interventions: modifications	#11b	Criteria for discontinuing or modifying allocated interventions for a given trial participant (eg, drug dose change in response to harms, participant request, or improving / worsening disease)	n/a
Interventions: adherance	#11c	Strategies to improve adherence to intervention protocols, and any procedures for monitoring adherence (eg, drug tablet return; laboratory tests)	n/a
Interventions: concomitant care	#11d	Relevant concomitant care and interventions that are permitted or prohibited during the trial	n/a
Outcomes	#12	Primary, secondary, and other outcomes, including the specific measurement variable (eg, systolic blood pressure), analysis metric (eg, change from baseline, final value, time to event), method of aggregation (eg, median, proportion), and time point for each outcome. Explanation of the clinical relevance of chosen efficacy and harm outcomes is strongly recommended	10

For peer review only

Participant timeline	#13	Time schedule of enrolment, interventions (including any run-ins and washouts), assessments, and visits for participants. A schematic diagram is highly recommended (see Figure)	12
Sample size	#14	Estimated number of participants needed to achieve study objectives and how it was determined, including clinical and statistical assumptions supporting any sample size calculations	10
Recruitment	#15	Strategies for achieving adequate participant enrolment to reach target sample size	6, 9
**Methods: Assignment of interventions (for controlled trials)**			
Allocation: sequence generation	#16a	Method of generating the allocation sequence (eg, computer-generated random numbers), and list of any factors for stratification. To reduce predictability of a random sequence, details of any planned restriction (eg, blocking) should be provided in a separate document that is unavailable to those who enrol participants or assign interventions	6
Allocation concealment mechanism	#16b	Mechanism of implementing the allocation sequence (eg, central telephone; sequentially numbered, opaque,	6

For peer review only

		sealed envelopes), describing any steps to conceal the sequence until interventions are assigned	
Allocation: implementation	#16c	Who will generate the allocation sequence, who will enrol participants, and who will assign participants to interventions	6
Blinding (masking)	#17a	Who will be blinded after assignment to interventions (eg, trial participants, care providers, outcome assessors, data analysts), and how	7
Blinding (masking): emergency unblinding	#17b	If blinded, circumstances under which unblinding is permissible, and procedure for revealing a participant's allocated intervention during the trial	n/a
**Methods: Data collection, management, and analysis**			
Data collection plan	#18a	Plans for assessment and collection of outcome, baseline, and other trial data, including any related processes to promote data quality (eg, duplicate measurements, training of assessors) and a description of study instruments (eg, questionnaires, laboratory tests) along with their reliability and validity, if known. Reference to where data collection forms can be found, if not in the protocol	10

For peer review only

Data collection plan: retention	#18b	Plans to promote participant retention and complete follow-up, including list of any outcome data to be collected for participants who discontinue or deviate from intervention protocols	n/a
Data management	#19	Plans for data entry, coding, security, and storage, including any related processes to promote data quality (eg, double data entry; range checks for data values). Reference to where details of data management procedures can be found, if not in the protocol	12
Statistics: outcomes	#20a	Statistical methods for analysing primary and secondary outcomes. Reference to where other details of the statistical analysis plan can be found, if not in the protocol	11
Statistics: additional analyses	#20b	Methods for any additional analyses (eg, subgroup and adjusted analyses)	11
Statistics: analysis population and missing data	#20c	Definition of analysis population relating to protocol non-adherence (eg, as randomised analysis), and any statistical methods to handle missing data (eg, multiple imputation)	n/a
**Methods: Monitoring**			
Data monitoring: formal committee	#21a	Composition of data monitoring committee (DMC); summary of its role and reporting structure; statement of whether it is independent from the sponsor and competing interests; and reference to where further	n/a

For peer review only

		details about its charter can be found, if not in the protocol. Alternatively, an explanation of why a DMC is not needed	
Data monitoring: interim analysis	#21b	Description of any interim analyses and stopping guidelines, including who will have access to these interim results and make the final decision to terminate the trial	12
Harms	#22	Plans for collecting, assessing, reporting, and managing solicited and spontaneously reported adverse events and other unintended effects of trial interventions or trial conduct	n/a
Auditing	#23	Frequency and procedures for auditing trial conduct, if any, and whether the process will be independent from investigators and the sponsor	12
**Ethics and dissemination**			
Research ethics approval	#24	Plans for seeking research ethics committee / institutional review board (REC / IRB) approval	6
Protocol amendments	#25	Plans for communicating important protocol modifications (eg, changes to eligibility criteria, outcomes, analyses) to relevant parties (eg, investigators, REC / IRBs, trial participants, trial registries, journals, regulators)	12

For peer review only

Consent or assent	#26a	Who will obtain informed consent or assent from potential trial participants or authorised surrogates, and how (see Item 32)	6
Consent or assent: ancillary studies	#26b	Additional consent provisions for collection and use of participant data and biological specimens in ancillary studies, if applicable	6
Confidentiality	#27	How personal information about potential and enrolled participants will be collected, shared, and maintained in order to protect confidentiality before, during, and after the trial	6, 12
Declaration of interests	#28	Financial and other competing interests for principal investigators for the overall trial and each study site	14
Data access	#29	Statement of who will have access to the final trial dataset, and disclosure of contractual agreements that limit such access for investigators	14
Ancillary and post trial care	#30	Provisions, if any, for ancillary and post-trial care, and for compensation to those who suffer harm from trial participation	n/a
Dissemination policy: trial results	#31a	Plans for investigators and sponsor to communicate trial results to participants, healthcare professionals, the public, and other relevant groups (eg, via publication, reporting in results databases, or other data sharing arrangements), including any publication restrictions	13

For peer review only

Dissemination policy: authorship	#31b	Authorship eligibility guidelines and any intended use of professional writers	14
Dissemination policy: reproducible research	#31c	Plans, if any, for granting public access to the full protocol, participant-level dataset, and statistical code	n/a

Appendices

Informed consent materials	#32	Model consent form and other related documentation given to participants and authorised surrogates	Suppl. file
Biological specimens	#33	Plans for collection, laboratory evaluation, and storage of biological specimens for genetic or molecular analysis in the current trial and for future use in ancillary studies, if applicable	n/a

None The SPIRIT Explanation and Elaboration paper is distributed under the terms of the Creative Commons Attribution License CC-BY-NC. This checklist can be completed online using https://www.goodreports.org/, a tool made by the EQUATOR Network in collaboration with Penelope.ai

For peer review only

BMJ Open

Increasing uptake of FIT colorectal screening: protocol for the TEMPO randomised controlled trial testing a suggested deadline and a planning tool

Journal:	*BMJ Open*
Manuscript ID	bmjopen-2022-066136.R2
Article Type:	Protocol
Date Submitted by the Author:	26-Apr-2023
Complete List of Authors:	Robb, Katie; University of Glasgow<br>Kotzur, Marie; University of Glasgow, Institute of Health & Wellbeing<br>Young, Ben; University of Glasgow<br>McCowan, Colin ; University of St. Andrews<br>Hollands, Gareth; University College London<br>Irvine, Audrey; NHS Tayside<br>MacDonald, Sara; University of Glasgow<br>McConnachie, Alex; University of Glasgow<br>O'carroll, Ronan; University of Stirling<br>O'Connor, Rory; University of Glasgow, Institute of Health and Wellbeing<br>Steele, Robert; University of Dundee, Surgery and Molecular Oncology
**Primary Subject Heading**:	Public health
Secondary Subject Heading:	Oncology
Keywords:	Gastrointestinal tumours < ONCOLOGY, PUBLIC HEALTH, PREVENTIVE MEDICINE

SCHOLARONE™
Manuscripts

Increasing uptake of FIT colorectal screening: protocol for the TEMPO randomised controlled trial testing a suggested deadline and a planning tool

Kathryn A. Robb*—School of Health and Wellbeing, University Of Glasgow, Glasgow, UK

Marie Kotzur—School of Health & Wellbeing, University Of Glasgow, Glasgow, UK

Ben Young—School of Health & Wellbeing, University Of Glasgow, Glasgow, UK

Colin McCowan—School of Medicine, University Of St. Andrews, St. Andrews, UK

Gareth J. Hollands—EPPI Centre, UCL Social Research Institute, University College London, London, UK

Audrey Irvine — Scottish Bowel Screening Centre, NHS Tayside, Dundee, UK

Sara Macdonald—School of Health & Wellbeing, University Of Glasgow, Glasgow, UK

Alex McConnachie— School of Health & Wellbeing, University Of Glasgow, Glasgow, UK

Ronan E. O'Carroll—Psychology, University of Stirling, Stirling, UK

Rory C. O'Connor— School of Health & Wellbeing, University Of Glasgow, Glasgow, UK

Robert J.C. Steele—School of Medicine, University of Dundee, Dundee, UK

* corresponding author: Prof Kathryn A. Robb| School of Health and Wellbeing | Admin Building | Gartnavel Royal Hospital | 1055 Great Western Road | Glasgow G12 0XH; Katie.robb@glasgow.ac.uk; +44 (0)141 211 0685.

Word count: 4460

For peer review only

Abstract

Introduction: Screening can reduce deaths from colorectal cancer (CRC). Despite high levels of public enthusiasm, participation rates in population CRC screening programmes internationally remain persistently below target levels. Simple behavioural interventions such as completion goals and planning tools may support participation among those inclined to be screened but who fail to act on their intentions. This study aims to evaluate the impact of: a) a suggested deadline for return of the test; b) a planning tool; and c) the combination of a deadline and planning tool on return of faecal immunochemical tests (FITs) for CRC screening.

Methods and analysis: A randomised controlled trial of 40,000 adults invited to participate in the Scottish Bowel Screening Programme will assess the individual and combined impact of the interventions. Trial delivery will be integrated into the existing CRC screening process. The Scottish Bowel Screening Programme mails FITs to people aged 50-74 with brief instructions for completion and return. Participants will be randomised to one of eight groups: 1) no intervention; 2) suggested deadline (1-week); 3) suggested deadline (2-weeks); 4) suggested deadline (4-weeks); 5) planning tool; 6) planning tool plus suggested deadline (1-week); 7) planning tool plus suggested deadline (2-weeks); 8) planning tool plus suggested deadline (4-weeks). The primary outcome is return of the correctly completed FIT at three months. To understand the cognitive and behavioural mechanisms and to explore the acceptability of both interventions, we will survey (n=2000) and interview (n=40) a subgroup of trial participants.

Ethics and dissemination: The study has been approved by the National Health Service South Central—Hampshire B Research Ethics Committee (ref. 19/SC/0369). The findings will be disseminated through conference presentations and publication in peer-reviewed journals. Participants can request a summary of the results.

Trial registration The trial is registered prospectively at clinicaltrials.gov (Identifier: NCT05408169, registration date: 07.06.2022).

For peer review only

Article summary

Strengths and limitations of this study

1. A suggested deadline for kit return and a FIT planning tool are highly cost-effective (if effective) interventions that could be easily implemented in routine practice, through adding a sentence to the invitation letter and including a sheet of paper, respectively.
2. A 2x4 factorial RCT design will allow us to test the individual and combined impact of two behavioural interventions on colorectal screening uptake.
3. Integrating the RCT delivery into the existing colorectal screening process will provide evidence of the feasibility of providing either or both within a well-established national screening programme.
4. Cross-sectional and qualitative evaluation research will provide further insight into the psychological mechanisms that underlie any intervention effects.

For peer review only - http://bmjopen.bmj.com/site/about/guidelines.xhtml

5. While the study supports key priorities within the UK Cancer Strategies to increase uptake of FIT colorectal screening, if the interventions are effective, this will lead to an increase in demand on follow up colonoscopy which currently has limited capacity.

Keywords (3-10) colorectal cancer; screening; faecal immunochemical test; deadline; planning; randomised controlled trial; qualitative interview

For peer review only

INTRODUCTION

Colorectal cancer (CRC) is the second leading cancer killer in Scotland and worldwide.[1,2] Screening has been shown to reduce deaths from CRC if enough people invited participate.[3,4] The challenge is that high uptake is hard to achieve, and remains persistently below the target levels internationally. For example, screening rates are 44% in Australia and 62% in the USA.[5,6] Following the introduction of the single-sample faecal immunochemical test (FIT) in 2017, screening uptake in Scotland has risen to 65%, thus exceeding the Scottish target rate of 60%,[7] but remains just below the internationally accepted European Union target uptake rate of at least 66%.[8] Although this is higher than the European population-based CRC screening programmes average of 25%,[8] one third of the population still do not screen and uptake is unevenly distributed across socioeconomic factors leading to health inequalities.[7]

Theoretical rationale

CRC screening uptake remains sub-optimal, despite high levels of public enthusiasm. In a Scottish survey, 85% of people reported intending to complete the FIT,[9] yet participation is 65%.[7] This suggests that apparent enthusiasm for the new FIT is not being translated into uptake. A major reason for this disparity may be that people are 'not getting round to it',[10] reflecting the well-recognised intention-behaviour gap and consistent with the observation that much of our behaviour is determined by factors other than conscious, deliberative control.[11-14] This disparity is consistent with previous work on 'inclined abstainers',[13] showing that a proportion of people are inclined to participate in screening but fail to do it. This explanation seems particularly likely for CRC screening which, unusually for a screening test, is self-completed at home. General open-ended intentions to act often fail to lead to the desired behaviour.[15] Our previous research has found that people often put off or forget to complete and return the FIT.[16] Additional support through prompting the setting of FIT completion goals and planning may better support participation among those inclined to complete the FIT but who might otherwise fail to act.

Goal setting

Goal setting, and particularly action-planning, can help initiate behaviour in those inclined to act. This is achieved by prompting a person to specifically plan when, where and how to act, which lessens the decision-making burden when that situation is encountered.[17-19] For example, a suggested deadline by which a task should be completed can be prompt to undertaking action planning. Currently, CRC screening invitations in Scotland provide detailed information about 'where' and 'how to act' but 'when to act' is left open, potentially reducing the likelihood that recipients will plan and act accordingly. In breast and cervical screening, providing a fixed appointment time increased uptake compared to an open invitation.[20] Similarly, in our recent qualitative study, women who attended breast and cervical screening but had not completed CRC screening described how the lack of an appointment facilitated the delay and forgetting of CRC screening: "If it's a bowel screening one, yep, put it somewhere and think 'Yes, I'll do that' and then forget about it because it doesn't have an appointment date. I think if something has an appointment date, you're forced to act." (54

For peer review only - http://bmjopen.bmj.com/site/about/guidelines.xhtml

years, not screened for CRC).[16(p.6)] Recent evidence suggests that both short (1-week) and long (3-week) deadlines achieve small but significant increases in FIT return.[21] Optimal deadline length, however, has yet to be determined. It is important to establish if a suggested deadline for FIT kit return can increase CRC screening participation compared to an open invitation in a population-based screening programme.

Planning support

Systematic reviews clearly demonstrate that interventions supporting people to plan how to enact a behaviour (including action and coping planning) are particularly suited to changing the behaviour of inclined abstainers.[12] Indeed, planning support interventions have been shown to be more effective than other approaches, such as self-monitoring or providing instructions,[22] and have high adherence rates.[23] Planning interventions are often based on implementation intentions which promote behaviour change using 'if-then' plans and have successfully reduced alcohol consumption and smoking, and increased cancer screening attendance and physical activity.[11,12,24,25] Previous research on implementation intention interventions for CRC screening has had limited success when it has relied on prepared statements of barriers ('if') and possible solutions ('then').[26] Encouraging people to form their own implementation intentions for CRC screening appears to produce clinically significant increases in uptake.[24] Planning tools (also called volitional help sheets [VHS]) emphasise this by requiring users to physically draw a line to link a barrier e.g. "If I am tempted to eat when I am anxious" with a solution e.g. "then I will reward myself when I do not overeat." to ensure user-engagement.[27(p.706)] The rationale is that the 'if' barriers or critical situations are made personalised and salient and, as a result, the 'then' response or solution comes automatically to mind.

The Medical Research Council's recently updated guidance on complex intervention development and evaluation recommends addressing questions beyond intervention effectiveness, such as "whether and how the intervention will be acceptable, implementable, cost effective, scalable, and transferable across contexts.".[28(p.2)] With a view to implementing a suggested deadline or planning tool at national scale, if effective, the acceptability of both interventions to CRC screening service users must be investigated.

Objectives

The overall aim of the TimE fraMe and Planning tOol (TEMPO) study is to evaluate the impact of a suggested deadline and planning tool on FIT return in the Scottish Bowel Screening Programme.

The study addresses six research questions:

1. Does providing a suggested deadline increase uptake of CRC screening?
2. Does providing a FIT planning tool increase uptake of CRC screening?
3. Does providing both a suggested deadline and a FIT planning tool increase uptake of CRC screening?
4. Does the length of the suggested deadline (one, two or four weeks) impact upon uptake of CRC screening?

For peer review only

5. What are the cognitive and behavioural mechanisms underlying any observed effect of providing a suggested deadline, a planning tool, or a combination of both?
6. How acceptable do participants find receiving a suggested deadline, a planning tool, or a combination of both?

METHODS AND ANALYSIS

The study employs a 2x4 factorial randomised controlled trial (RCT) design combined with cross-sectional quantitative and qualitative evaluation research with a sub-sample of trial participants. Figure 1 shows the CONSORT flow chart.

Study setting

This is a single-centre trial to be conducted in collaboration with the Scottish Bowel Screening Centre based in Dundee, Scotland, and integrated into the national CRC screening programme. In Scotland, colorectal screening is offered to people aged 50-74, every two years, by single-sample faecal immunochemical test (FIT).

Eligibility criteria

All people eligible for CRC screening in Scotland who have a Community Health Index Number can be included in this study.

Recruitment and allocation

Following established recruitment procedures,[29,30] working in partnership with the Scottish Bowel Screening Centre staff, 40,000 consecutive adults due to be sent a FIT will be randomised to one of eight groups (Figure 1) from 20.06.2022 over approximately 10 days.

The randomisation schedule will be computer-generated at the Robertson Centre for Biostatistics, University of Glasgow. The schedule will be stored in a secure area of the Robertson Centre for Biostatistics network, with no access for those developing the statistical analysis programs and transferred via a secure web portal to the Scottish Bowel Screening Centre team, who identify when people are to be invited for CRC screening. The IT system electronically generates the appropriate mailing packages to be sent out to each person. For this study, randomisation will be performed by the method of randomised permuted blocks of length 8, so that in every 8 packages sent, exactly one of each type will be included.

Blinding

The process of generating and mailing the routine screening invitation letters is fully automated and is conducted by a large mail-handling company. A data file containing contact details for the screening invitation letters is sent to the mail-handling company daily. The company will print screening invitation letters with suggested deadlines and add the planning tool in accordance with the participants' allocation within this study. The researchers, including those performing the analysis, will therefore be blind to the allocation of participants. The participants will not be informed of the experiment.

For peer review only - http://bmjopen.bmj.com/site/about/guidelines.xhtml

Interventions

There will be eight allocation groups (Figure 1): Group 1 – standard invitation (n = 5,000); Group 2 – 1-week deadline (n=5,000); Group 3 – 2-week deadline (n=5,000); Group 4 – 4-week deadline (n=5,000); Group 5 – planning tool (n=5,000); Group 6 – planning tool plus 1-week deadline (n=5,000); Group 7 – planning tool plus 2-week deadline (n=5,000); Group 8 – planning tool plus 4-week deadline (n=5,000).

Deadline intervention

Those allocated to the deadline intervention (groups 2-4 and 6-8) will receive a screening invitation letter with one of three suggested deadlines added (Table 1): 1 week, 2 weeks and 4 weeks. This choice was based on feedback from the Cancer Research UK Early Diagnosis Advisory Group, public and patient involvement representatives, and observation of kit return patterns in the Scottish Bowel Screening Programme. The deadline will be displayed centrally on the standard CRC screening invitation letter as a bold, highlighted sentence (Table 1). The wording *"or as soon as possible"* is intended to make clear that the deadline constitutes a suggested return date only, to avoid discouraging participants who are unable to meet the deadline. The existing invitation letter already states "*post it as soon as possible*" and the suggested deadline builds on this by providing a more explicit and specific goal. Furthermore, a standard reminder will be sent after 6 weeks without a deadline and so will provide a safety net for any potential participants who have missed the deadline in their first invitation letter.

Table 1 Deadline intervention arms

Intervention group	Suggested FIT return deadline	Deadline sentence
Groups 2 and 6	1-week deadline	Please return your kit within 1 week (by [insert date 10 days from posting date]) or as soon as possible.
Groups 3 and 7	2-week deadline	Please return your kit within 2 weeks (by [insert date 17 days from posting date]) or as soon as possible.
Groups 4 and 8	4-week deadline	Please return your kit within 4 weeks (by [insert date 31 days from posting date]) or as soon as possible.

Planning tool intervention

Those allocated to the planning tool intervention (groups 5-8) will receive a planning tool with their screening invitation. The planning tool will be presented on a separate single sheet of paper inserted into the CRC screening invitation envelope. It asks participants to identify "*concerns*" (i.e. barriers) they have with using the FIT from a list and to link them to a "*tip*" (i.e. solution) from an adjacent list to help them overcome this concern. The planning tool reads, *"Here are some tips that people find helpful to make the bowel screening kit even easier. Try drawing a line from any concern you have to a tip which might help you. You can draw as many or as few lines as you like. There are no right or wrong answers."* Concerns

For peer review only

reflect critical situations in conventional planning tool designs and tips reflect solutions (Armitage et al., 2014). Each concern and tip is accompanied by an image illustrating its meaning. Finally, participants will have the option to write their own concern or tip. While the planning tool instructions encourage people to use the planning tool, they do not suggest that participants must do so in order to take part in CRC screening. The specific listed concerns and tips are based on extensive development work involving consideration of existing theoretical and empirical literature, in-depth qualitative interviews, a survey of people who had been invited to complete a FIT, and co-design workshops to create a user-friendly planning tool with members of the public who have completed the FIT and others who have not completed the FIT.[31-33] The final planning tool was reviewed and approved by the Research Team, Patient and Public Involvement representatives, and a patient and public group of the Scottish Primary Care Network.
Table 2 displays the concerns and tips shown in the planning tool. The planning tool is presented in Supplementary file 1.

Table 2 Planning tool intervention—concerns and tips

Concerns	Tips
If I feel scared about bowel screening…	… then I'll read the instructions.
If I keep putting off using the kit…	… then I'll think this this kit could help save my life.
If I am not used to using a kit like this…	… then I'll put the kit by the toilet to remind me.
If I am worried what it might find…	… then I'll wash my hands after using the kit.
If I think using the kit is messy…	… then I'll tell myself that I'm responsible for my health.

Control group
As the trial has a factorial design, it follows that the control group for the assessment of the independent effect of each intervention is all those who did not receive the intervention. This is because any impact of one intervention impacts to an equal extent those who do and do not receive the other intervention. Therefore, groups 1 and 5 together make up the control group for the assessment of suggested deadlines and groups 1-4 make up the control group for assessment of the planning tool. Only participants in Group 1 will be sent a standard CRC screening invitation and FIT (without a suggested deadline or a planning tool) according to normal current practice. It is also important to note that three groups will receive both a planning tool and a suggested deadline, which will allow assessments of the combined effects of a suggested deadline and the planning tool compared to receiving a deadline only, a planning tool only, or neither.

Evaluation research of cognitive and behavioural mechanisms and acceptability
We will conduct two studies answering research questions 5 and 6.

Questionnaire survey
We will conduct a questionnaire-based case-control study to assess the cognitive and behavioural mechanisms underlying any effect of providing a suggested deadline for FIT kit return and a planning tool.

For peer review only - http://bmjopen.bmj.com/site/about/guidelines.xhtml

Recruitment. At least three months after the screening invitation, a subsample of trial participants will be identified from all eight groups, including people who returned (case) and did not return (control) their FIT, and people living in 40% most and least deprived neighbourhoods (based on SIMD quintiles). The subsample will be sent a postal invitation to complete and return a survey. The aim is to obtain responses from approximately 1,000 people who have returned the FIT (case) and 1,000 who have not returned (control) the FIT, across the eight groups. Based on previous work, we anticipate achieving a higher response rate from those returning the FIT and a lower response from those not returning the FIT, therefore approximately 11,872 questionnaires will be mailed, as shown in Figure 1.[32] A reminder questionnaire will be sent to those who have not returned the questionnaire after three weeks.

Materials and procedure. A brief questionnaire will include items to assess cognitive and behavioural mechanisms, e.g. action and coping planning,[17] that were previously adapted for the CRC screening context. [19, 32] It will also include a theory-informed acceptability measure that has been subject to a pre-validation method but does not yet have fully established psychometric properties,[34] as well as demographic characteristics. Participants will be asked if they are willing to participate in future research to assist recruitment for qualitative interviews.

Qualitative interviews

We will conduct a qualitative interview study, adopting a phenomenological approach to explore participants' experiences of receiving a FIT with and without the interventions, and explore acceptability of the interventions.

Recruitment. Interview participants will be sampled from survey participants who have indicated willingness to participate in further research. We will recruit approximately 40 participants and will aim to achieve a balance of participants who have and have not returned the FIT, men and women, from across the CRC screening invitation age range (50-74 years), from both deprived and affluent neighbourhoods (based on SIMD), and from the trial intervention groups. The estimated sample size is based on the principles of achieving data saturation and what is pragmatic, yet informative.

Procedure. Potential participants will be contacted to request participation in a semi-structured qualitative interview conducted face-to-face, by telephone or video-call. Interviews will last approximately 30-60 minutes, and topic guides (Supplementary file 2) will be used. Participants will be offered £30 towards travel and expenses.

Outcomes

Our primary outcome is the proportion of FITs returned correctly completed to be tested by the CRC screening laboratory providing a positive or negative result, within three months of the FIT being sent to a person. The return and successful testing of any replacement FITs that were sent to people after the first FIT at study baseline will be treated as a FIT return. As secondary outcomes, we will evaluate cognitive and behavioural mechanisms and explore the acceptability of both interventions.

For peer review only - http://bmjopen.bmj.com/site/about/guidelines.xhtml

Sample size

This 2x4 factorial RCT involves the assessment of two interventions, so each will be assessed at a significance level of 2.5%, to preserve an overall Type I error rate of 5%.

The assessment of the effect of the planning tool will involve the comparison of two groups of 20,000 participants. At a significance level of 2.5%, this gives 90% power to detect an increase in uptake rates from 65% to 66.8%. Alternatively, if the intervention increases uptake rates from 65% to 68%, the 97.5% confidence interval for the difference in uptake rates will have a width of ±1.06%.

The assessment of the effect of suggested deadlines for FIT kit return will involve four groups of 10,000 participants. Comparing each suggested deadline with the control group (no deadline) at a significance level of 0.83% (one third of 2.5%), there will be 90% power to detect increases in uptake rates from 65% to 67.63%. If an intervention increases uptake from 65% to 68%, the 99.17% confidence interval for the difference in uptake rates will have a width of ±1.76%.

Survey. The study is a case-control study (1,000 cases, 1,000 controls). The study will have 90% power at a 5% significance level to detect an odds ratio for being a case of 1.146 per standard deviation increase in a continuous predictor variable; for a binary predictor with 50% prevalence, the study will have 90% power to detect an odds ratio for being a case of 1.345; for a binary predictor with a prevalence of 10%, the study will have 90% power to detect an odds ratio for being a case of 1.662.

Trial data collection and management

For the purposes of this study, two additional variables will be added to the Scottish Bowel Screening Programme invitation data file: i) a unique identifier for each participant; and ii) a variable coding for the allocated condition. The pseudonymised participant data including FIT kit return data will be transferred from the Scottish Bowel Screening Centre in Dundee to the Robertson Centre for Biostatistics at the University of Glasgow using a secure file transfer protocol.

Data analysis plan

Primary and secondary outcome measures will be summarised using descriptive statistics.

Primary outcome

The primary analysis will use a logistic regression model, including a binary variable for whether the planning tool was provided, and a four-level categorical variable denoting which deadline was suggested. Age, sex, and area deprivation (measured by the Scottish Index of Multiple Deprivation [SIMD][35]) will be included in the model, to improve model specification. The main effects of each intervention will be reported as an odds ratio, with a confidence interval (CI) and p-value. For the estimate of the effect of the planning tool, a 97.5% CI will be reported. For estimates of the effect of alternative suggested deadlines, relative to no deadline, 99.17% CIs will be used. Subsequent analyses will model the

For peer review only - http://bmjopen.bmj.com/site/about/guidelines.xhtml

interaction between the two interventions, to assess whether the interventions have independent effects or are synergistic. We shall also model interactions between each intervention effect and age, sex, and deprivation, to assess whether the interventions have different effects for subgroups of the population. Assessing uptake by deprivation is important given the disparities in screening uptake by deprivation (53% most deprived versus 73% least deprived;[7]) which any intervention could reduce or exacerbate.[36]

Survey analysis. Descriptive statistics, Chi-square tests, ANOVA and logistic regression will be used to examine cognitive and behavioural mechanism differences between the intervention and control groups and their association with FIT kit return.

Qualitative analysis. The interviews will be audio-recorded, transcribed verbatim, checked by the interviewer and analysed using the Framework Method.[37] This method facilitates systematic, rigorous and transparent data management, providing an audit trail from raw data to final themes. We will use NVivo software to undertake coding, manage and apply a working analytical framework, generate framework matrices and summarise and interpret data. This method will adopt a combined deductive and inductive approach, guided by the behavioural mechanisms targeted by the interventions (e.g. action and coping planning),[17] as well as constructs within the theoretical framework of acceptability,[38] whilst also allowing other experiences and any unintended intervention effects to be explored. BY will lead the analysis with support from KAR, MK and SM. Interpretation of findings may also involve the wider co-investigator group, including the project Patient & Public Representatives, for improved credibility of findings. In addition, the results from the survey will be used to inform the analysis of the qualitative interviews and vice versa to determine whether synthesising the two data sources can provide unique insights which would not be apparent by analysing the data independently. Calls to the Scottish Bowel Screening Programme helpline relating to the suggested deadline for FIT kit return and planning tool will also be monitored via a call report form including date, time and nature of query as an additional assessment of acceptability.

Participant timeline

This protocol describes a three-month project as shown in Figure 2.

Study management and monitoring

This trial combines two individually funded studies with the same Principal Investigator. The trial will be managed by the co-investigator teams of both studies including the Principal Investigator, academic co-investigators, key researchers, and public and patient involvement representatives who will oversee trial conduct and progress together. Data management, participant confidentiality and the conduct of all trial staff will adhere to the protocol (Version 1.4, 06.09.2021 or subsequent approved versions), Good Clinical Practice guidelines, and the Data Protection Act 2018. An audit trail of all documentation and data collection will be kept in an electronic study site file to facilitate monitoring by the research team and the sponsor. This trial is sponsored by the NHS Greater Glasgow and Clyde. Amendments to the protocol will be notified to the sponsor and the responsible Research Ethics Committee for their approval. The sponsor may audit the trial at any time and without notice.

For peer review only

Stopping criteria

The CRC screening invitations and kits will be posted as one batch over approximately 10 days. There are no stopping criteria for recruitment.

Harms

This is a low-risk trial providing people eligible for the Scottish Bowel Screening Programme with additional written information. While we recognise that providing a suggested deadline or a planning tool may increase the burden of completing the FIT, we will ensure that participants: a) are not disadvantaged if they cannot meet the suggested deadline for returning the FIT; and b) are under no obligation to use the planning tool in order to complete CRC screening. We will be able to evaluate the additional burden of each intervention in the questionnaire and interview evaluation studies.

Public and patient involvement

The study is supported by two patient and public representatives (PPRs) who have provided input throughout the programme of research. The PPRs were involved in the design of the study, the creation of materials to support the recruitment and join the co-investigator meetings to discuss study progress. At the end of the study, the PPRs will review a plain language summary of the study findings which we will make available to the interview participants.

Intervention materials for this study, i.e. the planning tool and the screening invitation letters including the three suggested deadlines, have been developed in workshops and focus groups and have received feedback from the PPRs, the Cancer Research UK Early Diagnosis Advisory Group, and the PPRs of the Scottish Primary Care Research network.

DISCUSSION

The purpose of this study is to increase uptake of FIT colorectal screening using two behavioural interventions. The study will evaluate the impact of a suggested deadline, a planning tool, and the combination of a deadline and a planning tool on return of FITs. This research is important because colorectal cancer is the second most common cause of cancer death globally [1] but uptake remains below target levels.

The study builds on existing theoretical and empirical evidence [11-17; 27] and is the first attempt to test the individual and combined impact of a deadline and a co-designed planning tool within the real-world setting of a national screening programme. In addition, this study will provide evidence on the most effective timeframe for a suggested deadline as well as the cognitive and behavioural mechanisms that underlie any intervention effects. Finally, the study will investigate the acceptability of providing either intervention to those invited to participate in CRC screening. If effective, the interventions will be highly cost effective. A simple change to the wording of the colorectal screening programmes' invitation letters (deadline) and the inclusion of a sheet of paper (planning tool) will be all that is required to immediately see the benefits of increased uptake and subsequent earlier diagnosis of colorectal cancer.

For peer review only

The study has a number of potential limitations. While the study supports key priorities within the UK Cancer Strategies to increase uptake of FIT colorectal screening, if the interventions are effective, this will lead to an increase in demand on follow up colonoscopy which currently has limited capacity. The primary outcome is return of a kit after 3 months rather than 6 months as used by the Scottish Bowel Screening Programme. This was because the study was severely delayed by the COVID-19 pandemic and the follow up was consequently reduced. It is unclear whether the results would be generalisable to screening programmes beyond the UK.The secondary outcomes examining the cognitive and behavioural mechanisms underlying any observed effects and the acceptability of the interventions will only be assessed after three months. Participants may not recall in detail their experience of receiving the deadline and/or the planning tool. Assessing the potential mechanisms immediately before and after test completion my have provided greater insight but this design risks contaminating the intervention itself. Our retrospective approach therefore provides the most appropriate option in this applied setting.

ETHICS AND DISSEMINATION

Ethical approval was granted by the UK National Health Service South Central – Hampshire B Research Ethics Committee (REC ref. no. 19/SC/0369). Written informed consent will not be obtained before inclusion in the trial to maintain ecological validity and to avoid selection bias. This is a common approach in comparable trials performed in the context of national screening programmes, [29, 30] without which it may not be viable to perform robust evaluations leading to evidence-based service improvements. There is very low risk of the interventions causing harm to participants and individuals will not be identifiable from trial data. Consent for the questionnaire survey will be implied through the completion of the questionnaire. Written informed consent to participate in a qualitative interview will be taken by the interviewer (Supplementary file 3). The research will be carried out in accordance with the World Medical Association Declaration of Helsinki (1964) and its revisions (Tokyo [1975], Venice [1983], Hong Kong [1989], South Africa [1996] and Edinburgh [2000]).

The results from this study will be presented at national and international conferences and written up for publication in peer reviewed journals. The study is expected to produce two manuscripts: i) primary findings of the RCT and ii) the findings of the secondary evaluation. The co-authors have a track record of public and media engagement in research. They will work closely with the University Of Glasgow Press Office to disseminate the findings to the public. Study participants will receive a summary of the findings if they request this.

DECLARATIONS

Acknowledgements

We thank the PPRs, Mary Cameron and Lucy J. Robertson, for their continued support of this research, the patient and public involvement group of the Scottish Primary Care Research Network for their feedback on the intervention materials, and Catherin McKay of

For peer review only - http://bmjopen.bmj.com/site/about/guidelines.xhtml

ListenThinkDraw who provided illustrative services during the development of the planning tool and images for the final version.

Author contributions

KAR conceived both the planning tool and deadline interventions. KAR and MK designed the intervention procedures, study materials and evaluation with input from BY, SM, REOC, RCOC, AI, CMC, AM, GJH and RJCS. AM conducted the sample size calculations and designed the statistical analyses. KAR submitted the original application for ethical approval and MK and BY drafted and managed approval amendments. KAR, BY and MK drafted the protocol manuscript. All co-authors read and approved the final manuscript.

Funding

This study is supported by an Early Diagnosis Advisory Group (EDAG) Project Grant from Cancer Research UK (C9227/A27877 - Increasing uptake of faecal immunochemical test (FIT) bowel screening: trial of providing a suggested deadline for FIT kit return, PI KAR, Co-investigators: CMC, AM, GJH, RJCS) and by a Response Mode Grant from the Scottish Chief Scientist Office (HIPS/17/23 - Increasing uptake of bowel cancer screening: development of a FIT planning support tool, PI: KAR, Co-investigators: SM, REOC, RCOC, AI, RJCS, MK). The funders have no involvement in the study design, data collection or analysis, or the writing and publication of reports of the study. All researchers involved in this study were independent of the funder.

Competing interests

The authors declare that they have no competing interests.

Consent for publication

Not applicable

Availability of data and materials

Not applicable

For peer review only

REFERENCES

1. Ferlay J, Colombet M, Soerjomataram I, Parkin DM, Piñeros M, Znaor A, Bray F. Cancer statistics for the year 2020: An overview. Int J Cancer. 2021;149:778-789.
2. Public Health Scotland. Cancer Mortality in Scotland: Annual update to 2021. [Internet]. 2022. Available from https://publichealthscotland.scot/media/15989/2022-10-25-cancer-mortality-report.pdf
3. Lin JS, Perdue LA, Henrikson NB, Bean SI, Blasi PR. Screening for Colorectal Cancer: Updated Evidence Report and Systematic Review for the US Preventive Services Task Force. JAMA. 2021;325(19):1978–1998.
4. Randel KR, Schult AL, Botteri E, Hoff G, Bretthauer M, Ursin G, Natvig E, Berstad P, Jørgensen A, Sandvei PK, Olsen ME, Frigstad SO, Darre-Næss O, Norvard ER, Bolstad N, Kørner H, Wibe A, Wensaas KA, de Lange T, Holme Ø. Colorectal Cancer Screening With Repeated Fecal Immunochemical Test Versus Sigmoidoscopy: Baseline Results From a Randomized Trial. Gastroenterology. 2021 Mar;160(4):1085-1096.e5.
5. Australian Institute of Health and Welfare National Bowel Cancer Screening Program monitoring report 2022. 2022; catalogue number CAN 148, AIHW, Australian Government.
6. White A, Thompson TD, White MC, et al. Cancer screening test use—United States, 2015. *MMWR Morb Mortal Wkly Rep* 2017;66(8):201.
7. Public Health Scotland. Scottish Bowel Screening Programme Statistics: For the period of invitations between May 2019 and April 2021. [Internet]. 2022. [cited 2022 June 23] Available at https://publichealthscotland.scot/media/11605/2022-02-08-bowel-screening-publication-report.pdf.
8. Senore C, Basu P, Anttila A, et al. Performance of colorectal cancer screening in the European Union Member States: data from the second European screening report. *Gut* 2019;68(7):1232-44.
9. Chambers JA, Callander AS, Grangeret R, et al. Attitudes towards the Faecal Occult Blood Test (FOBT) versus the Faecal Immunochemical Test (FIT) for colorectal cancer screening: Perceived ease of completion and disgust. *BMC Cancer* 2016;16(1):96-102.
10. Lo SH, Waller J, Wardle J, et al. Comparing barriers to colorectal cancer screening with barriers to breast and cervical screening: A population–based survey of screening-age women in Great Britain. *J Med Screen* 2013;20(2):73-79.
11. Gollwitzer PM, Sheeran P. Implementation intentions and goal achievement: A meta-analysis of effects and processes. In: Advances in experimental social psychology, Vol. 38. San Diego: Elsevier Academic Press Inc. 2006:69-119.
12. Kwasnicka D, Presseau J, White M, et al. Does planning how to cope with anticipated barriers facilitate health-related behaviour change? A systematic review. *Health Psychol Rev* 2013;7(2):129-145.
13. Orbell S, Sheeran P. 'Inclined abstainers': A problem for predicting health-related behaviour. Br J Soc Psychol 1998;37(2):151-165.
14. Hollands GJ, Marteau TM, Fletcher PC. Non-conscious processes in changing health-related behaviour: A conceptual analysis and framework. *Health Psychol Rev* 2016;10(4):381–394.

For peer review only - http://bmjopen.bmj.com/site/about/guidelines.xhtml

15. Webb T, Sheeran P. Does changing behavioural intentions engender behaviour change: A meta-analysis of the experimental evidence. *Psychol Bull 2006;132:*249-268.
16. Kotzur M, McCowan C, Macdonald S, et al. Why colorectal screening fails to achieve the uptake rates of breast and cervical cancer screening: a comparative qualitative study. *BMJ Qua Saf* 2020;29(6):482-90.
17. Gollwitzer PM. Implementation intentions: Strong effects of simple plans. *Am Psychol* 1999;54(7): 493.
18. Michie S, Dormandy E, Marteau TM. Increasing screening uptake amongst those intending to be screened: the use of action plans. Patient Educ Couns 2004;55(2):218-22.
19. Presseau J, Johnston M, Heponiemi T, et al. Reflective and automatic processes in health care professional behaviour: a dual process model tested across multiple behaviours. *Ann Behav Med* 2014;48(3), 347-358.
20. Duffy SW, Myles JP, Maroni R, et al. Rapid review of evaluation of interventions to improve participation in cancer screening services. *J Med Screen* 2017;24(3): 127-145.
21. Lieberman, A., Gneezy A, Berry E. The effect of deadlines on cancer screening completion: a randomized controlled trial. *Sci Rep* 2021;11(1):13876.
22. Dombrowski SU, Sniehotta FF, Avenell A, et al. Identifying active ingredients in complex behavioural interventions for obese adults with obesity-related co-morbidities or additional risk factors for co-morbidities: A systematic review. *Health Psychol Rev* 2012;6(1):7-32.
23. O'Connor RC, Ferguson E, Scott F, et al. A randomised controlled trial of a brief psychological intervention to reduce repetition of self-harm in patients admitted to hospital following a suicide attempt. *Lancet Psychiatry* 2017;4(6):451-60.
24. Neter E, Stein N, Barnett-Griness O, et al. From the Bench to Public Health: Population-level Implementation Intentions in Colorectal Cancer Screening. *Am J Prev Med* 2014;46(3): 273-280.
25. Rutter DR, Steadman L, Quine L. An implementation intentions intervention to increase uptake of mammography. *Ann Behav Med* 2006;32(2):127-34.
26. Lo SH, Good A, Sheeran P, et al. Preformulated implementation intentions to promote colorectal cancer screening: A cluster-randomized trial. *Health Psychol* 2014;33(9):998-1002.
27. Armitage CJ, Norman P, Noor M, et al. Evidence that a very brief psychological intervention boosts weight loss in a weight loss program. *Behav Ther* 2014;45(5):700–707.
28. Skivington K, Matthews L, Simpson SA, et al. A new framework for developing and evaluating complex interventions: Update of Medical Research Council guidance. *BMJ* 2021;374.
29. Libby, G, Bray, J, Champion, J, et al. Pre-notification increases uptake of colorectal cancer screening in all demographic groups: a randomized controlled trial. *J Med Screen* 2011;8,24-29.
30. O'Carroll RE, Chambers JA, Brownlee L, et al. Anticipated regret to increase uptake of colorectal cancer screening (ARTICS): A randomised controlled trial. *Soc Sci Med.* 2015;142:118-127.

For peer review only - http://bmjopen.bmj.com/site/about/guidelines.xhtml

31. Kotzur M, Macdonald S, O'Connor R, et al. Identifying barriers and solutions to self-completed FIT bowel screening to develop a volitional help-sheet. 33rd Annual Conference of the European Health Psychology Society; 2019 September 03-07; Dubrovnik, Croatia.
32. Kotzur M, Macdonald S, O'Carroll RE, et al. What are common barriers and helpful solutions to colorectal cancer screening? A cross-sectional survey to develop intervention content for a planning support tool; BMJ Open 2022;12:e062738. .
33. Robb KA. Achieving solutions to inequalities in cancer screening. Cancer Research UK/UK Society for Behavioural Medicine Cancer Special Interest Group Behavioural Science Webinar; 2022 June 07; virtual seminar.
34. Sekhon, M., Cartwright, M. & Francis, J.J. Development of a theory-informed questionnaire to assess the acceptability of healthcare interventions. BMC Health Serv Res 22, 279 (2022).
35. Scottish Government. Scottish Index of Multiple Deprivation 2020. [Internet]. [cited 2022 May 25]. Available from https://www.gov.scot/collections/scottish-index-of-multiple-deprivation-2020/
36. Lorenc T, Petticrew M, Welch V, et al. What types of interventions generate inequalities? Evidence from systematic reviews. *J Epidemiol Community Health* 2013;67:190-193.
37. Ritchie J, Spencer L. Qualitative data analysis for applied policy research. In Ritchie J, Spencer L, Bryman A et al. eds. Analysing Qualitative Data. London, UK: Routledge 1994:173-194.
38. Sekhon M, Cartwright M, Francis JJ. Acceptability of healthcare interventions: an overview of reviews and development of a theoretical framework. *BMC Health Serv Res* 2017;17(1):88.

For peer review only - http://bmjopen.bmj.com/site/about/guidelines.xhtml

FIGURE TITLES AND LEGENDS

Figure 1 CONSORT flow chart

Figure 2 Participant timeline
Note. * denotes data collection end points

For peer review only

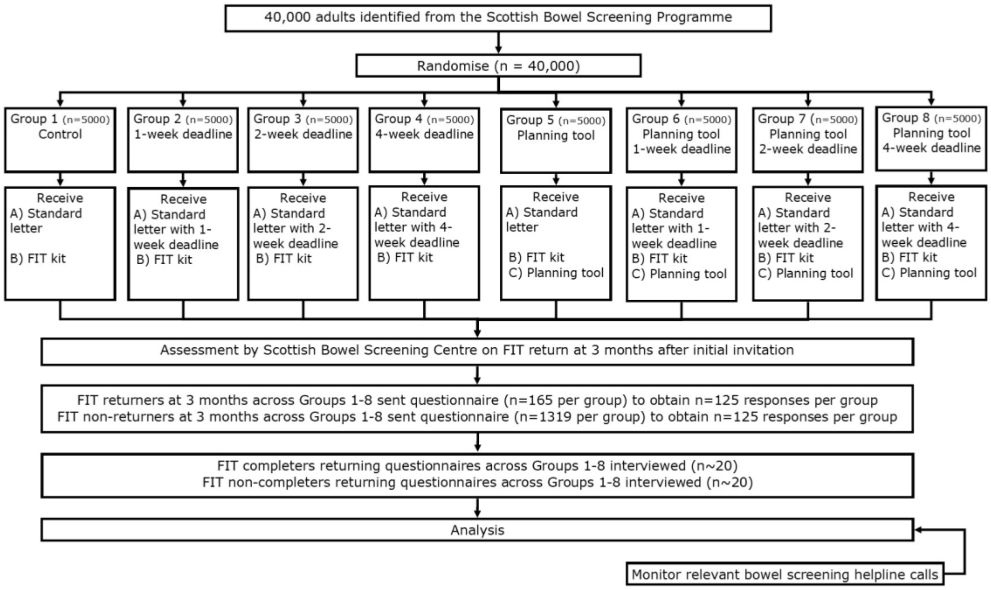

Figure 1 CONSORT flow chart

297x209mm (150 x 150 DPI)

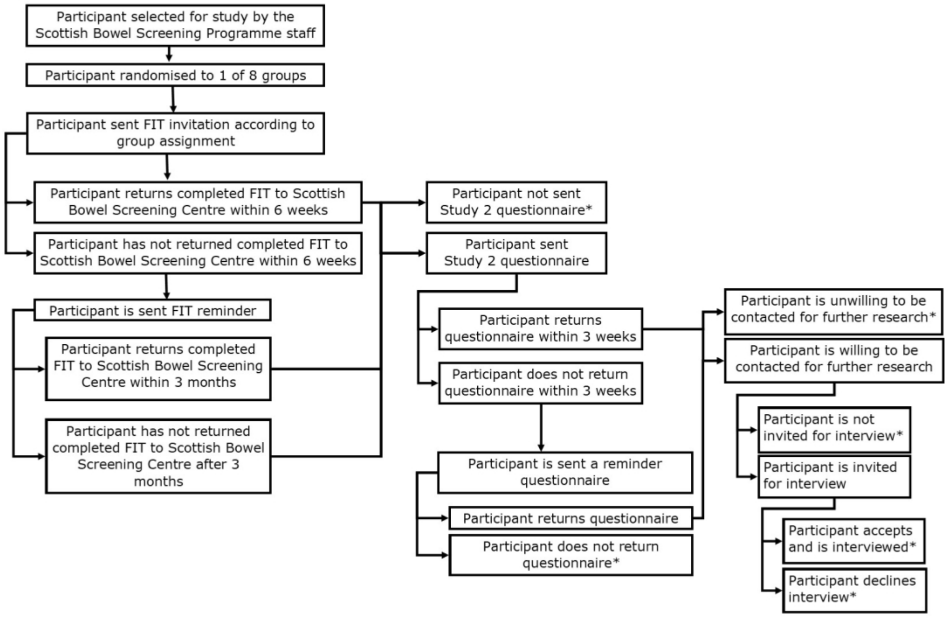

Figure 2 Participant timeline

297x209mm (150 x 150 DPI)

TIPS FOR USING YOUR KIT

Here are some tips that people find helpful to make the bowel screening kit even easier. Try drawing a line from any concern you have to a tip which might help you. You can draw as many or as few lines as you like. There are no right or wrong answers.

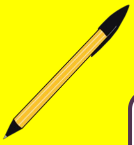

Concerns

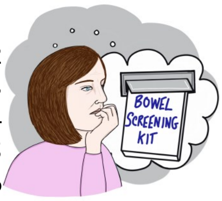

If I feel scared about bowel screening... ●

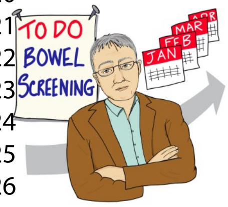

If I keep putting off using the kit... ●

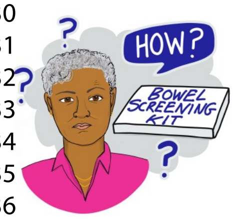

If I am not used to using a kit like this... ●

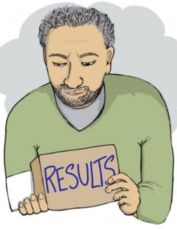

If I am worried what it might find... ●

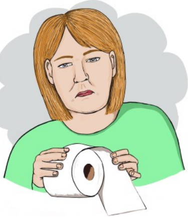

If I think using the kit is messy... ●

Do you have another concern? Please write it here: *If I…* ●

____

____

____

Tips

● … then I'll read the instructions.

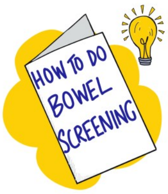

● … then I'll think that this kit could help save my life.

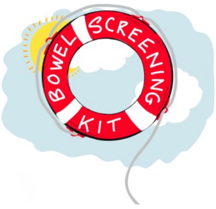

● … then I'll put the kit by the toilet to remind me.

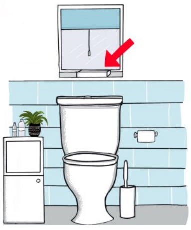

● … then I'll wash my hands after using the kit.

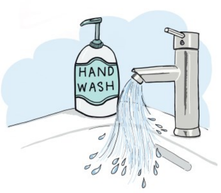

● ...then I'll tell myself that I'm responsible for my health.

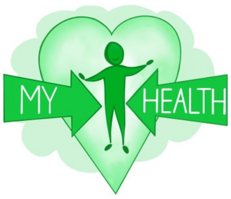

● Got a better tip? Please write it here: ...then *I'll…*

____

____

____

For peer review only - http://bmjopen.bmj.com/site/about/guidelines.xhtml

Control group i **(no deadline, no planning sheet)**
FIT completers and FIT non-completers

Topic Guide

People's views on bowel screening

1. Experience of completing the FIT kit

A few months ago you received a test kit like this in the post. Thinking back, can you tell me exactly what you did?

- Did you have a clear plan about if/when/where/how you would do the test?
  - was it something you decided/did automatically/without thinking?
- Was there anything that you found hard or difficult about doing the test?
  - *Prompt with barriers (forgetting, disgusting, embarrassing, worry, afraid of results, tempting fate, lack of time, unsure how to do)*
    - *Did you plan to overcome these?*

2. Acceptability of deadlines

It has been suggested that giving people a suggested deadline for returning the bowel screening kit would be helpful. For example, people would be asked to return the kit within 1 week or 2 weeks or 4 weeks.

- Do you think this would be acceptable? Would particular deadlines be more or less acceptable/effective? What would be your preference?
- Would you like or dislike being a given a suggested deadline?
- Do you think it is fair to peole to give a suggested deadline for returning the bowel screening kit?
- Is it clear to you how being given a suggested deadline for returning the kit would be helpful in completing the kit?

3. Acceptability of planning sheet

It has been suggested that asking people to link their concerns with tips for the bowel screening kit would be helpful. For example, people would be given a page with concerns and tips for using their bowel screening kit and asked to draw a line from a concern they have to a tip which might help them.

- **Do you think this would be acceptable? Would particular concerns/ tips be more or less acceptable/effective? What would be your preference?**
- **Would you like or dislike being being asked to link concerns and tips for your bowel screening kit?**
- **Do you think it is fair to ask peole to link concerns and tips for their bowel screening kit?**
- **Is it clear to you how being asked to link concerns and tips for your bowel screening kit would be helpful in completing the kit? Would you find it helpful, in what way?**

For peer review only - http://bmjopen.bmj.com/site/about/guidelines.xhtml

Intervention groups **ii-iv (1-, 2-, 4-week deadline, no planning sheet)**
FIT completers and FIT non-completers

Topic Guide

People's views on bowel screening

1. Experience of completing the FIT kit

   A few months ago you received a test kit like this in the post. Thinking back, can you tell me exactly what you did?

   - Did you have a clear plan about if/when/where/how you would do the test?
     - was it something you decided/did automatically/without thinking?
   - Was there anything that you found hard or difficult about doing the test?
     - *Prompt with barriers (forgetting, disgusting, embarrassing, worry, afraid of results, tempting fate, lack of time, unsure how to do)*
       - *Did you plan to overcome these?*

2. Acceptability of deadlines

   Did you notice you were asked to return the kit by a certain time (1week/2 weeks/4 weeks) ?

   - Did you think this was acceptable? Would particular deadlines be more or less acceptable/effective? What would be your preference?
   - Did you like or dislike being a given a suggested deadline?
   - Do you think it is fair to peole to give a suggested deadline for returning the bowel screening kit?
   - Is it clear to you how being given a suggested deadline for returning the kit would be helpful in completing the kit? Did you find it helpful, in what way?

3. **Acceptability of planning sheet**

   It has been suggested that asking people to link their concerns with tips for the bowel screening kit would be helpful. For example, people would be given a page with concerns and tips for using their bowel screening kit and asked to draw a line from a concern they have to a tip which might help them.

   - **Do you think this would be acceptable? Would particular concerns/ tips be more or less acceptable/effective? What would be your preference?**
   - **Would you like or dislike being being asked to link concerns and tips for your bowel screening kit?**
   - **Do you think it is fair to ask peole to link concerns and tips for their bowel screening kit?**
   - **Is it clear to you how being asked to link concerns and tips for your bowel screening kit would be helpful in completing the kit? Would you find it helpful, in what way?**

For peer review only - http://bmjopen.bmj.com/site/about/guidelines.xhtml

Intervention group v (no deadline, with a planning sheet)
FIT completers and FIT non-completers

Topic Guide

People's views on bowel screening

1. **Experience of completing the FIT kit**

   A few months ago you received a test kit like this in the post. Thinking back, can you tell me exactly what you did?

   - Did you have a clear plan about if/when/where/how you would do the test?
     - was it something you decided/did automatically/without thinking?
   - Was there anything that you found hard or difficult about doing the test?
     - *Prompt with barriers (forgetting, disgusting, embarrassing, worry, afraid of results, tempting fate, lack of time, unsure how to do)*
       - *Did you plan to overcome these?*

2. **Acceptability of deadlines**

   It has been suggested that giving people a suggested deadline for returning the bowel screening kit would be helpful. For example, people would be asked to return the kit within 1 week or 2 weeks or 4 weeks.

   - Do you think this would be acceptable? Would particular deadlines be more or less acceptable/effective? What would be your preference?
   - Would you like or dislike being a given a suggested deadline?
   - Do you think it is fair to peole to give a suggested deadline for returning the bowel screening kit?
   - Is it clear to you how being given a suggested deadline for returning the kit would be helpful in completing the kit?

3. **Acceptability of planning sheet**

   Did you notice you were given a page with tips for using your bowel screening kit?

   - Did you think this was acceptable? Would particular concerns/ tips be more or less acceptable/effective? What would be your preference?
   - Did you like or dislike being asked to link concerns and tips for your bowel screening kit?
   - Do you think it is fair to ask peole to link concerns and tips for their bowel screening kit?
   - Is it clear to you how being asked to link concerns and tips for your bowel screening kit would be helpful in completing the kit? Did you find it helpful, in what way?

For peer review only - http://bmjopen.bmj.com/site/about/guidelines.xhtml

Intervention groups vi-viii (1-, 2-, 4-week deadline, with a planning sheet)
FIT completers and FIT non-completers

Topic Guide

People's views on bowel screening

1. **Experience of completing the FIT kit**
   A few months ago you received a test kit like this in the post. Thinking back, can you tell me exactly what you did?
   - Did you have a clear plan about if/when/where/how you would do the test?
     - was it something you decided/did automatically/without thinking?
   - Was there anything that you found hard or difficult about doing the test?
     - *Prompt with barriers (forgetting, disgusting, embarrassing, worry, afraid of results, tempting fate, lack of time, unsure how to do)*
       - *Did you plan to overcome these?*

2. **Acceptability of deadlines**
   Did you notice you were asked to return the kit by a certain time (1week/2 weeks/4 weeks) ?
   - Did you think this was acceptable? Would particular deadlines be more or less acceptable/effective? What would be your preference?
   - Did you like or dislike being a given a suggested deadline?
   - Do you think it is fair to peole to give a suggested deadline for returning the bowel screening kit?
   - Is it clear to you how being given a suggested deadline for returning the kit would be helpful in completing the kit? Did you find it helpful, in what way?

3. **Acceptability of planning sheet**
   Did you notice you were given a page with tips for using your bowel screening kit?
   - Did you think this was acceptable? Would particular concerns/ tips be more or less acceptable/effective? What would be your preference?
   - Did you like or dislike being asked to link concerns and tips for your bowel screening kit?
   - Do you think it is fair to ask peole to link concerns and tips for their bowel screening kit?
   - Is it clear to you how being asked to link concerns and tips for your bowel screening kit would be helpful in completing the kit? Did you find it helpful, in what way?

For peer review only - http://bmjopen.bmj.com/site/about/guidelines.xhtml

University of Glasgow
School of Health & Wellbeing

NHS SCOTLAND

Mental Health and Wellbeing, Gartnavel Royal Hospital, Administration Building, 1st Floor, 1055 Great Western Road, Glasgow, G12 0XH

CONSENT FORM

Title of project: **People's views on bowel screening – interview study**

Name of Researchers: Dr Ben Young and Prof Katie Robb

Please initial box

1. I confirm that I have read and understand the information sheet Version 1.3 dated 5.8.2022 for the above study. ☐
2. I have had the opportunity to consider the information, ask questions and have had these answered satisfactorily ☐
3. I agree to the interview being audio-recorded and to its contents being used for research purposes. ☐
4. I understand that my participation is voluntary and that I am free to withdraw at any time, without giving any reason, without my medical care or legal rights being affected. ☐
5. I understand that my medical notes and data collected from the study may be looked at by regulatory authorities or by persons from NHS Scotland where it is relevant to my taking part in this study. I agree to these persons having access to this information. ☐
6. I agree to take part in the above study. ☐

____________________	____________________	____________________
Name of participant	Date	Signature
____________________	____________________	____________________
Researcher	Date	Signature

1 original for participant, 1 original for researcher

For peer review only - http://bmjopen.bmj.com/site/about/guidelines.xhtml

Reporting checklist for protocol of a clinical trial.

Based on the SPIRIT guidelines.

Instructions to authors

Complete this checklist by entering the page numbers from your manuscript where readers will find each of the items listed below.

Your article may not currently address all the items on the checklist. Please modify your text to include the missing information. If you are certain that an item does not apply, please write "n/a" and provide a short explanation.

Upload your completed checklist as an extra file when you submit to a journal.

In your methods section, say that you used the SPIRITreporting guidelines, and cite them as:

Chan A-W, Tetzlaff JM, Gøtzsche PC, Altman DG, Mann H, Berlin J, Dickersin K, Hróbjartsson A, Schulz KF, Parulekar WR, Krleža-Jerić K, Laupacis A, Moher D. SPIRIT 2013 Explanation and Elaboration: Guidance for protocols of clinical trials. BMJ. 2013;346:e7586

For peer review only

		Reporting Item	Page Number
**Administrative information**			
Title	#1	Descriptive title identifying the study design, population, interventions, and, if applicable, trial acronym	1

Trial registration	#2a	Trial identifier and registry name. If not yet registered, name of intended registry	2
Trial registration: data set	#2b	All items from the World Health Organization Trial Registration Data Set	n/a
Protocol version	#3	Date and version identifier	n/a
Funding	#4	Sources and types of financial, material, and other support	14
Roles and responsibilities: contributorship	#5a	Names, affiliations, and roles of protocol contributors	14
Roles and responsibilities: sponsor contact information	#5b	Name and contact information for the trial sponsor	12
Roles and responsibilities: sponsor and funder	#5c	Role of study sponsor and funders, if any, in study design; collection, management, analysis, and interpretation of data; writing of the report; and the decision to submit the report for publication, including whether they will have ultimate authority over any of these activities	14
Roles and responsibilities: committees	#5d	Composition, roles, and responsibilities of the coordinating centre, steering committee, endpoint adjudication committee, data management team, and	n/a

For peer review only

		other individuals or groups overseeing the trial, if applicable (see Item 21a for data monitoring committee)	
**Introduction**			
Background and rationale	#6a	Description of research question and justification for undertaking the trial, including summary of relevant studies (published and unpublished) examining benefits and harms for each intervention	4
Background and rationale: choice of comparators	#6b	Explanation for choice of comparators	8
Objectives	#7	Specific objectives or hypotheses	5
Trial design	#8	Description of trial design including type of trial (eg, parallel group, crossover, factorial, single group), allocation ratio, and framework (eg, superiority, equivalence, non-inferiority, exploratory)	6
**Methods: Participants, interventions, and outcomes**			
Study setting	#9	Description of study settings (eg, community clinic, academic hospital) and list of countries where data will be collected. Reference to where list of study sites can be obtained	6

For peer review only

Eligibility criteria	#10	Inclusion and exclusion criteria for participants. If applicable, eligibility criteria for study centres and individuals who will perform the interventions (eg, surgeons, psychotherapists)	6
Interventions: description	#11a	Interventions for each group with sufficient detail to allow replication, including how and when they will be administered	7
Interventions: modifications	#11b	Criteria for discontinuing or modifying allocated interventions for a given trial participant (eg, drug dose change in response to harms, participant request, or improving / worsening disease)	n/a
Interventions: adherance	#11c	Strategies to improve adherence to intervention protocols, and any procedures for monitoring adherence (eg, drug tablet return; laboratory tests)	n/a
Interventions: concomitant care	#11d	Relevant concomitant care and interventions that are permitted or prohibited during the trial	n/a
Outcomes	#12	Primary, secondary, and other outcomes, including the specific measurement variable (eg, systolic blood pressure), analysis metric (eg, change from baseline, final value, time to event), method of aggregation (eg, median, proportion), and time point for each outcome. Explanation of the clinical relevance of chosen efficacy and harm outcomes is strongly recommended	10

For peer review only

Participant timeline	#13	Time schedule of enrolment, interventions (including any run-ins and washouts), assessments, and visits for participants. A schematic diagram is highly recommended (see Figure)	12
Sample size	#14	Estimated number of participants needed to achieve study objectives and how it was determined, including clinical and statistical assumptions supporting any sample size calculations	10
Recruitment	#15	Strategies for achieving adequate participant enrolment to reach target sample size	6, 9
**Methods: Assignment of interventions (for controlled trials)**			
Allocation: sequence generation	#16a	Method of generating the allocation sequence (eg, computer-generated random numbers), and list of any factors for stratification. To reduce predictability of a random sequence, details of any planned restriction (eg, blocking) should be provided in a separate document that is unavailable to those who enrol participants or assign interventions	6
Allocation concealment mechanism	#16b	Mechanism of implementing the allocation sequence (eg, central telephone; sequentially numbered, opaque,	6

For peer review only

		sealed envelopes), describing any steps to conceal the sequence until interventions are assigned	
Allocation: implementation	#16c	Who will generate the allocation sequence, who will enrol participants, and who will assign participants to interventions	6
Blinding (masking)	#17a	Who will be blinded after assignment to interventions (eg, trial participants, care providers, outcome assessors, data analysts), and how	7
Blinding (masking): emergency unblinding	#17b	If blinded, circumstances under which unblinding is permissible, and procedure for revealing a participant's allocated intervention during the trial	n/a
**Methods: Data collection, management, and analysis**			
Data collection plan	#18a	Plans for assessment and collection of outcome, baseline, and other trial data, including any related processes to promote data quality (eg, duplicate measurements, training of assessors) and a description of study instruments (eg, questionnaires, laboratory tests) along with their reliability and validity, if known. Reference to where data collection forms can be found, if not in the protocol	10

For peer review only

Data collection plan: retention	#18b	Plans to promote participant retention and complete follow-up, including list of any outcome data to be collected for participants who discontinue or deviate from intervention protocols	n/a
Data management	#19	Plans for data entry, coding, security, and storage, including any related processes to promote data quality (eg, double data entry; range checks for data values). Reference to where details of data management procedures can be found, if not in the protocol	12
Statistics: outcomes	#20a	Statistical methods for analysing primary and secondary outcomes. Reference to where other details of the statistical analysis plan can be found, if not in the protocol	11
Statistics: additional analyses	#20b	Methods for any additional analyses (eg, subgroup and adjusted analyses)	11
Statistics: analysis population and missing data	#20c	Definition of analysis population relating to protocol non-adherence (eg, as randomised analysis), and any statistical methods to handle missing data (eg, multiple imputation)	n/a

Methods: Monitoring

Data monitoring: formal committee	#21a	Composition of data monitoring committee (DMC); summary of its role and reporting structure; statement of whether it is independent from the sponsor and competing interests; and reference to where further	n/a

For peer review only

		details about its charter can be found, if not in the protocol. Alternatively, an explanation of why a DMC is not needed	
Data monitoring: interim analysis	#21b	Description of any interim analyses and stopping guidelines, including who will have access to these interim results and make the final decision to terminate the trial	12
Harms	#22	Plans for collecting, assessing, reporting, and managing solicited and spontaneously reported adverse events and other unintended effects of trial interventions or trial conduct	n/a
Auditing	#23	Frequency and procedures for auditing trial conduct, if any, and whether the process will be independent from investigators and the sponsor	12
**Ethics and dissemination**			
Research ethics approval	#24	Plans for seeking research ethics committee / institutional review board (REC / IRB) approval	6
Protocol amendments	#25	Plans for communicating important protocol modifications (eg, changes to eligibility criteria, outcomes, analyses) to relevant parties (eg, investigators, REC / IRBs, trial participants, trial registries, journals, regulators)	12

For peer review only

Consent or assent	#26a	Who will obtain informed consent or assent from potential trial participants or authorised surrogates, and how (see Item 32)	6
Consent or assent: ancillary studies	#26b	Additional consent provisions for collection and use of participant data and biological specimens in ancillary studies, if applicable	6
Confidentiality	#27	How personal information about potential and enrolled participants will be collected, shared, and maintained in order to protect confidentiality before, during, and after the trial	6, 12
Declaration of interests	#28	Financial and other competing interests for principal investigators for the overall trial and each study site	14
Data access	#29	Statement of who will have access to the final trial dataset, and disclosure of contractual agreements that limit such access for investigators	14
Ancillary and post trial care	#30	Provisions, if any, for ancillary and post-trial care, and for compensation to those who suffer harm from trial participation	n/a
Dissemination policy: trial results	#31a	Plans for investigators and sponsor to communicate trial results to participants, healthcare professionals, the public, and other relevant groups (eg, via publication, reporting in results databases, or other data sharing arrangements), including any publication restrictions	13

For peer review only

Dissemination policy: authorship	#31b	Authorship eligibility guidelines and any intended use of professional writers	14
Dissemination policy: reproducible research	#31c	Plans, if any, for granting public access to the full protocol, participant-level dataset, and statistical code	n/a

Appendices

Informed consent materials	#32	Model consent form and other related documentation given to participants and authorised surrogates	Suppl. file
Biological specimens	#33	Plans for collection, laboratory evaluation, and storage of biological specimens for genetic or molecular analysis in the current trial and for future use in ancillary studies, if applicable	n/a

None The SPIRIT Explanation and Elaboration paper is distributed under the terms of the Creative Commons Attribution License CC-BY-NC. This checklist can be completed online using https://www.goodreports.org/, a tool made by the EQUATOR Network in collaboration with Penelope.ai

For peer review only
